# Experimental Evidence of Functional Group-Dependent Effects of Tree Diversity on Soil Fungi in Subtropical Forests

**DOI:** 10.3389/fmicb.2018.02312

**Published:** 2018-10-09

**Authors:** Christina Weißbecker, Tesfaye Wubet, Guillaume Lentendu, Peter Kühn, Thomas Scholten, Helge Bruelheide, François Buscot

**Affiliations:** ^1^Department of Soil Ecology, Helmholtz-Centre for Environmental Research - UFZ, Halle, Germany; ^2^Institute of Biology, Leipzig University, Leipzig, Germany; ^3^German Centre for Integrative Biodiversity Research (iDiv) Halle-Jena-Leipzig, Leipzig, Germany; ^4^Department of Ecology, University of Kaiserslautern, Kaiserslautern, Germany; ^5^Chair of Soil Science and Geomorphology, University of Tübingen, Tübingen, Germany; ^6^Institute of Biology, Martin Luther University Halle Wittenberg, Halle, Germany

**Keywords:** BEF-China, experimental forest, forest biodiversity experiment, fungal functional groups, host preference, metagenomics, mycorrhizal fungi, soil

## Abstract

Deconvoluting the relative contributions made by specific biotic and abiotic drivers to soil fungal community compositions facilitates predictions about the functional responses of ecosystems to environmental changes, such as losses of plant diversity, but it is hindered by the complex interactions involved. Experimental assembly of tree species allows separation of the respective effects of plant community composition (biotic components) and soil properties (abiotic components), enabling much greater statistical power than can be achieved in observational studies. We therefore analyzed these contributions by assessing, via pyrotag sequencing of the internal transcribed spacer (ITS2) rDNA region, fungal communities in young subtropical forest plots included in a large experiment on the effects of tree species richness. Spatial variables and soil properties were the main drivers of soil fungal alpha and beta-diversity, implying strong early-stage environmental filtering and dispersal limitation. Tree related variables, such as tree community composition, significantly affected arbuscular mycorrhizal and pathogen fungal community structure, while differences in tree host species and host abundance affected ectomycorrhizal fungal community composition. At this early stage of the experiment, only a limited amount of carbon inputs (rhizodeposits and leaf litter) was being provided to the ecosystem due to the size of the tree saplings, and persisting legacy effects were observed. We thus expect to find increasing tree related effects on fungal community composition as forest development proceeds.

## Introduction

**FIGURE 1:**
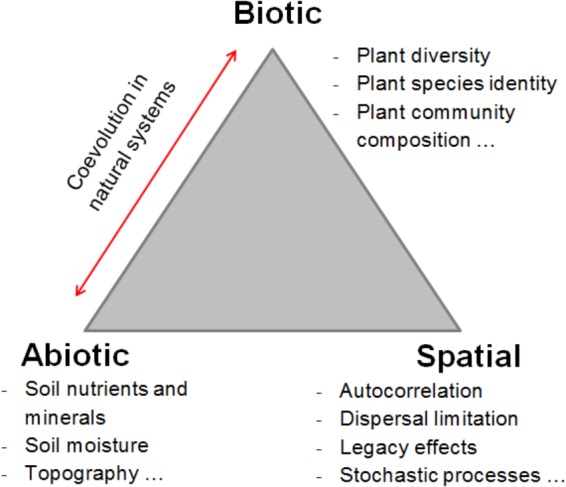
Conceptual overview of the main drivers of soil fungal community assembly.

Soil fungi are a highly diverse group of organisms (possibly including several million species; Blackwell, 2011; Taylor et al., 2014), providing many ecosystem services such as organic matter decomposition, element cycling, plant nutrition and plant protection (van der Heijden et al., 2015). They can be assigned to functional guilds based on the primary classes of resources they exploit (Nguyen et al., 2016a), and the composition of their communities is governed by multiple, strongly interacting abiotic, biotic and spatial variables (**Figure 1**). Unraveling the relative contributions of these potential drivers to fungal community composition will greatly facilitate predictions about ecosystem functioning in response to environmental changes, particularly reductions in plant diversity.

Land plants and fungi share intimately associated natural histories (Buscot, 2015). From the onset of the colonization of terrestrial habitats, plants have gained crucial support from arbuscular mycorrhizal (AM) fungi (Humphreys et al., 2010), which provide their plant symbionts with substantial proportions of their phosphorus requirements (Smith and Smith, 2011). Saprotrophic fungi evolved to be the most prominent group of microorganisms capable of decomposing complex plant residues (Floudas et al., 2012), and their descendants include ectomycorrhizal (EcM) fungi. The latter mainly decompose nitrogen-rich polymers, and trade nitrogen and micronutrients against photosynthates with their plant symbionts, thereby greatly extending the plants’ ability to acquire both mineral and organic soil resources (Bruns and Shefferson, 2004). Pathogenic fungi can substantially impair plant growth and fecundity (Zeilinger et al., 2016), or even destroy large populations of their hosts, but they also play important roles in maintaining plant diversity and mediating plant succession in forest ecosystems (Gilbert, 2002).

In natural ecosystems, biotic and abiotic components often show strong interdependencies as plant communities coevolve with the abiotic soil matrix, and they interact to affect the physico-chemical conditions of the soils (Augusto et al., 2002; Ayres et al., 2009; Condit et al., 2013; Zemunik et al., 2016). Hence in observational studies it is methodologically difficult to assess the contributions made by each specific factor to the development of ecosystems and their responses to environmental changes (Peay et al., 2010; Martínez-García et al., 2015; Schappe et al., 2017). Correlations between biotic and abiotic factors must be taken into account in order to allow inferences about causal relationships underlying community assemblies and confirmation of insights gained by the multitude of observational studies conducted on regional (e.g., Gao et al., 2015; Martínez-García et al., 2015; Urbanova et al., 2015; Tedersoo et al., 2016; Schappe et al., 2017) and global (Tedersoo et al., 2014; Davison et al., 2015; Prober et al., 2015) scales. While Tedersoo et al. (2014) and Prober et al. (2015) postulated that plant and fungal alpha diversity are independent on the global scale (EcM fungi being an exception), several studies have detected significant regional relationships between these variables (tropics: Peay et al., 2013; subtropics: Liang et al., 2015; temperate: Martínez-García et al., 2015; Urbanova et al., 2015; Tedersoo et al., 2016). In terms of community composition, Prober et al. (2015) found a strong correlation between AM fungal and plant beta-diversity in grasslands. Moreover, regional correlations between subtropical tree and AM fungal communities and between tropical tree and non-AM fungal communities have been found by, respectively, Liang et al. (2015) and Schappe et al. (2017). In contrast, EcM fungal communities have been reported to be related to host identity (Ishida et al., 2007; Tedersoo et al., 2008, 2010) and host richness and abundance (Tedersoo et al., 2014), at both host species and higher phylogenetic levels, including host genera (Gao et al., 2013) and families (Tedersoo et al., 2012).

Experimental assembly of host plant species makes it possible to separate the effects of plant community composition (biotic components) and soil properties (abiotic components) on the plant-fungus relationships and associated functional responses of ecosystems. Information about microbial communities in experimental forests will increase in the coming years as many experimental forest platforms have been established recently (Verheyen et al., 2016; Grossman et al., 2018; Paquette et al., 2018). Currently, though, published studies on soil fungal diversity are scarce. To the best of our knowledge, the only published studies on soil fungi conducted in tree biodiversity experiments are those by Nguyen et al. (2016b) and Tedersoo et al. (2016). Nguyen et al. (2016b) focused on relationships among tree and EcM fungal and saprotroph communities in young temperate-boreal forests in the Cloquet IDENT experiment (United States), which includes both American and European tree species. These were assembled in 12 monoculture and 4 six-species mixture plots (replicated four times in a block design, resulting in a total of 64 plots). They detected significant correlations between the beta-diversities of the trees and both the EcM and saprotroph soil fungal communities, but no significant correlations between fungal and tree alpha diversities. Tedersoo et al. (2016) found context-dependent tree diversity and species identity effects on soil fungi among tree experimental forest sites in Finland (the Satakunta experiment) and Estonia. Tree richness was positively correlated with soil fungal groups in Estonia and with EcM fungi in Finland. Communities of soil biota were generally driven by spatial eigenvectors in Finland and soil properties in Estonia. Furthermore, fungal richness was most strongly associated with herb cover and tree basal area.

Here, we extend these findings by reporting the results of a study of fungal communities in plots in a young large-scale subtropical experimental forest in China planted with 1, 2, 4, 8, or 16 native tree species included in a Biodiversity-Ecosystem Functioning experiment designated BEF-China (Bruelheide et al., 2014). We assessed the contributions made by biotic (tree community variables), abiotic (soil properties and topography) and spatial information to the soil fungal richness and community patterns of the four main fungal functional groups: saprotrophic, pathogenic, AM and EcM fungi.

The interplay between plant-driven and abiotic processes and soil fungal communities is likely to vary in strength among fungal functional groups, since they differ in their degree of association with particular plant species. We hypothesized that AM and EcM fungi would show strong, but distinct, correlations with both plant community composition and abiotic soil properties. This is because both groups associate directly with plant roots and the soil matrix, but the ratio of host to symbiont diversity differs between the two types of mycorrhiza (Buscot, 2015). In contrast, necrotrophic parasites and saprotrophic fungi do not depend directly on living plants (Zeilinger et al., 2016), and we expected communities of these groups to be more strongly affected by abiotic soil properties than by the plant community.

## Materials and Methods

### Sampling Site

The samples analyzed in this study were collected from plots hosting 1–16 native subtropical tree species at the BEF-China site (Bruelheide et al., 2014), which was established in 2009 on a hillside in Southeast China, Jiangxi Province (29°07′26.0″N 117°54′29.0″E). The climate there is subtropical with warm wet summers and cold dry winters. January and July are the coldest and warmest months, with mean temperatures of 0.4 and 34.2°C, respectively. The mean annual temperature is 17.4°C and mean annual rainfall amounts to 1635 mm (Yang et al., 2013). Before 2008, the site was in forestry use and hosted an approximately 20-year-old mixed planted stand of trees of the economically important timber species *Pinus massoniana* (EcM) and *Cunninghamia lanceolata* (AM), which were clear-cut directly before establishment of the experiment. Monoculture plots of both tree species were present in the BEF-China experimental plantation scheme but were not included in the sampling design of the present study. The planted forest plots are located in the hill altitudinal zone, spanning elevations from 105 to 275 m a.s.l. and varying considerably in inclination, with an average slope of 25° (Scholten et al., 2017). The soils are mainly Cambisols and Cambisol derivatives, falling into the reference soil groups Regosols, Cambisols, Acrisols, and Gleysols (IUSS Working Group WRB, 2014), with Cambisols and Regosols on ridges, spurs and crests, Cambisols and Acrisols along slopes and colluvic Cambisols and Gleysols occurring predominantly on foot slopes and in valleys (Scholten et al., 2017). Soil samples collected for this study had pH values (H_2_O) of 4.1–5.6, total nitrogen contents of 0.08–0.31%, carbon to nitrogen ratios of 10–24, effective cation exchange capacities of 35–91 μmol g^-1^ soil and base saturation values of 6–92%.

A broken-stick design determined the experimental planting schemes of the 31 forest plots investigated here, i.e., the set of 16 native subtropical tree species was repeatedly sub-divided into subsets of eight, four, two, and one species to establish communities with lower diversity levels (**Supplementary Figure S1**). Each of the 16 tree species was represented once at each diversity level (monocultures, and mixtures of 2, 4, 8, and 16 tree species) and less diverse plots were nested within more diverse plots (Bruelheide et al., 2014). The total tree species pool has equal numbers of AM- and EcM-forming tree species, but lower diversity plots do not necessarily have equal proportions of AM and ECM trees (see **Supplementary Figure S1**). Each forest plot covers 25.8 m × 25.8 m, and tree species compositions were randomly assigned to plots. In each plot, 400 trees were planted at 1.29 m spacing, in a regular grid with assigned species being randomly allocated planting positions within the plot. We took samples for this study in October 2011 after the third growing season following planting. At this time the mean total tree height ranged from 52 to 301 cm depending on tree species (Li et al., 2014). Before sampling for our study, the herb layer was removed by weeding.

### Soil Sampling

In October 2011, we randomly selected five trees per species in each plot (where possible) for sampling of soil in their root zones. Thus we planned to collect five replicate samples of soil in root zones of all 16 tree species at each diversity level (400 samples in total). However, tree mortality prevented collection of six samples in *Castanopsis eyrei* root zones (five in the four-tree species plot and one in the 16-tree species plot), resulting in a final number of 394 samples.

Soil samples were collected within 7 days. Loose stones and litter were removed from the soil surface and the upper 10 cm of the mineral soil was sampled, by removing four cores (6 cm in diameter and 10 cm deep) at points 20–30 cm from each selected tree trunk in cardinal compass directions using an auger. The four soil cores obtained from the root zone of each selected tree were mixed, sieved (2 mm mesh size) and homogenized to form a composite soil sample. Two 15 g subsamples were immediately flash-frozen in liquid nitrogen for molecular analysis. One was stored permanently at -80°C as a backup. The other was freeze-dried for 48 h and subsequently stored at -80°C in a vacuum-sealed plastic bag containing silica gel prior to transportation to the processing laboratory in Germany. Freeze-dried soil samples were transported by airplane within 4 days, following the recommendation of Weißbecker et al. (2017), and immediately stored at -80°C until required for molecular analysis.

### Soil Chemical Analysis

Soil samples were air-dried, and a 50 g subsample of each sample was ground with a ball mill. The pH of sieved samples (<2 mm) resuspended in 25 ml double-distilled water was measured potentiometrically using a Sentix 81 electrode (WTW, Weilheim, Germany). Total organic carbon (*C*_tot_) and total nitrogen (*N*_tot_) contents of ground samples were measured using a Vario El III CN-analyzer (Elementar, Hanau, Germany). Because of the acidic soil conditions, no inorganic carbon was present, so *C*_tot_ represented the soil organic carbon content. The sieved soil samples were percolated with an unbuffered 1 M NH_4_Cl solution and the effective cation exchange capacity (CEC) of the extracts was measured with a DV 5300 inductively coupled plasma atomic emission spectrometer (PerkinElmer). Soil moisture content was assessed from water loss after freeze-drying.

### Nucleic Acid Extraction and Multiplexed Amplicon Pyrosequencing

Microbial DNA was extracted with a PowerSoil^®^ htp 96 Well Soil DNA Isolation Kit, RNA using a PowerSoil^®^ Total RNA Isolation Kit (MO BIO Laboratories Inc., Carlsbad, CA, United States). When using the first of these kits, 0.25 g samples of freeze-dried soil were extracted twice. When using the second kit, 1 g samples of freeze-dried soil were re-wetted with 1 ml clean water for 5 min before extraction. After RNA extraction, an RNA PowerSoil^®^ DNA Elution Accessory Kit was used according to the manufacturer’s instructions. A negative control with no soil was included in each batch of samples subjected to nucleic acid extraction. Initially, we intended to produce a cDNA dataset based on the extracted RNA as reported by, for example, Baldrian et al. (2012). However, we did not succeed in generating high quality sequences based on cDNA.

The nucleic acid extracts were quantified with a NanoDrop ND-8000 spectrophotometer (Peqlab, Germany). Fungal ITS rDNA amplicon libraries were generated using the fungal-specific ITS1F primer (Gardes and Bruns, 1993) containing Roche 454 pyrosequencing adaptor B, the universal primer ITS4 (White et al., 1990), Roche 454 pyrosequencing adaptor A and a sample-specific multiplex identifier sequence (MID). The ITS region has been proposed as an universal fungal barcode in metagenomic studies (Schoch et al., 2012) but it has also been reported to lack optimal resolving power for AM fungi (Stockinger et al., 2010). Nevertheless, Berruti et al. (2017) found similar patterns of diversity in AM fungal communities assessed by means of an ITS2 and an AM fungal specific 18S primer pair. The community-structuring effects of location and environment could be resolved correctly by the ITS2 targeting primers. Similar numbers of AM fungal operational taxonomic units (OTUs) have been found in Panamanian rainforest soils using fungal ITS and AM fungus-specific primers (Schappe et al., 2017). We amplified the ITS region sequences by PCR using 50 μl reaction mixtures containing 10 ng DNA template in 1 μl extraction buffer, 25 μl GoTaq Green Master Mix (Promega, Mannheim, Germany) and 1 μl of a solution providing 25 pmol of each of the ITS region-specific primers, as described by Wubet et al. (2012). All samples were amplified in triplicate. The PCR replicates were pooled, then purified using a gel extraction kit (Qiagen, Hilden, Germany). DNA concentrations of the purified amplicon products were measured using a Cary Eclipse fluorescence spectrophotometer (Agilent Technologies, Waldbronn, Germany). Equimolar pools of c. 60 sample amplicons were produced and processed according to instructions supplied with the GS FLX+ sequencing kit (Roche, Mannheim, Germany). The sequencing plate was divided into four lanes and one processed amplicon library pool was assigned to each lane. The amplicons were then sequenced by unidirectional pyrosequencing from the ITS4 ends using a Roche GS-FLX+ 454 pyrosequencer (Roche, Mannheim, Germany) at the Department of Soil Ecology, Helmholtz Centre of Environmental Research (UFZ, Halle, Germany).

### Bioinformatics Analysis

Multiple levels of sequence processing and quality filtering were applied using an in-house metabarcode analysis pipeline for grid engines based mainly on the MOTHUR (version 1.39.5, Schloss et al., 2009) and OBITools (version 1.2.11, Boyer et al., 2016) software suites. Initially, sequences with any barcode mismatches or four primer mismatches were removed. All primer and barcode sequences were discarded. Sequences with any ambiguous bases or homopolymers exceeding eight nucleotides were removed. Flows were denoised and reads were trimmed, using FlowClus (Gaspar and Thomas, 2015), to uniform 360 bp long read fragments spanning the ITS2 and the 5.8S rRNA gene. Chimeric reads were detected and removed from each sample using the UCHIME algorithm as implemented in MOTHUR (Edgar et al., 2011). Dereplicated quality-filtered sequences were sorted by decreasing abundance and clustered into OTUs using the vsearch algorithm (version 2.4.4, Rognes et al., 2016) with a sequence similarity threshold of 97%. Representative (the most abundant) sequences for each OTU were taxonomically assigned based on reference sequences from the UNITE database (version v7_2, Kõljalg et al., 2013) using the naïve Bayesian classifier (Wang et al., 2007), as implemented in MOTHUR, at a consensus threshold of 60%. The sequences identified as fungal were further classified against the full version of the unite.v7_2 database augmented with non-fungal eukaryotic ITS sequences retrieved from GenBank to improve taxonomic annotation and detect non-target OTUs. In addition, taxonomic assignments of the first 2500 OTUs were manually refined by inspection of the first 15 INSDC database blast search results. Assignments with *E*-values smaller than eˆ-50 were assumed to be reliable and sequence similarity thresholds of 75, 80, 85, 90, and 95% were applied for class, order, family, genus and species classifications, respectively. Putative functions were annotated using the FUNGuild fungal database (version 1.1, Nguyen et al., 2016a). Functional annotations were further refined using information accessible through the APSnet search engine of the American Phytopathological Society^1^ and the MycoBank database (Robert et al., 2013).

### Statistical Analysis

Fungal OTUs with at least four sequence reads were included in the statistical analyses; singletons, doubletons and tripletons were discarded. Non-metric multidimensional scaling (NMDS) ordinations based on 30 random starts were calculated from the abundant fungal OTU dataset (containing at least four sequences) and the original dataset containing all OTUs. Procrustes correlation analysis conducted on both ordinations revealed that fungal community composition was not significantly affected by the presence or absence of rare fungal and potentially artificial OTUs (Procrustes correlation coefficient = 0.9987, *p* < 0.001). Procrustes analysis was carried out applying the protest function (Peres-Neto and Jackson, 2001) of the vegan package (Oksanen et al., 2013). Zhan et al. (2014) found OTUs generated by pyrosequencing containing more than three sequences to be highly reproducible between sequence runs whereas the reproducibility of OTUs containing three (tripletons), two (doubletons) or one (singletons) sequence read(s) was drastically lower.

Statistical analyses were performed using R version 3.4.2 (R Core Team, 2014). The data matrices for taxonomic information, environmental variables measured and OTU abundances were merged using the phyloseq package (McMurdie and Holmes, 2013) to facilitate further splitting of the dataset into data pertaining to each of the fungal functional groups under consideration. For the individual fungal functional group analyses, samples were included in the statistical analysis only if an arbitrary read count of 20 (215 samples), 40 (178 samples), 210 (190 samples) and 250 (361 samples) was met for AM, pathogenic, EcM and saprotrophic fungi, respectively. These sequence thresholds correspond to approximately a tenth of the maximum read count for the respective functional groups in a sample. Due to missing tree community data, three samples had to be excluded from the statistical analysis.

We applied linear regression analyses to determine the contributions of tree community variables, soil properties, topography and spatial variables to fungal alpha and beta-diversity relationships. The tree species of the selected tree at each sampling position and its eight nearest neighbors were recorded. The tree community variables assessed included the following: tree species richness, Shannon and Simpson diversity indices of the trees, abundance and richness of EcM and AM trees, tree species identity, their mycorrhizal (ecto vs. arbuscular) type and tree community composition. The abiotic variables included the following soil properties: pH; total nitrogen (*N*_tot_), total carbon (*C*_tot_), and moisture contents; *C*:*N* ratio; effective cation exchange capacity (CEC); and base saturation (BS); all of these are important indicators of soil fertility (Lincoln et al., 2014; Scholten et al., 2017; Bünemann et al., 2018). In addition, two major topographical variables (altitude and slope) were taken into consideration because the experimental site is located on steep hills. The GPS coordinates of sampling locations were included in the statistical analysis as pairwise sampling distances or spatial eigenvectors.

All analyses were carried out at plot level. For all samples taken from the root zone of the same tree species in a plot, fungal read information was summed for richness analysis, and Hellinger-transformed fungal abundance counts were averaged. Due to the sequence thresholds applied, not all statistical sampling units contained the sequence information from five core replicates (per tree species per plot). While most samples were retained for the analysis of saprotrophic fungi, the number of core replicates was nearly evenly distributed from one to five sampling cores for the remaining fungal groups, AM, EcM and plant pathogenic fungi (**Supplementary Table S1**).

### Fungal Alpha Diversity Analysis

Species richness information in the fungal count data was derived using the vegan package (Oksanen et al., 2013) and regressed against the square root of the number of reads it was based upon. The resulting fungal richness residuals were included in model calculations. This approach is an alternative method for sequence normalization which is applied to avoid severe loss of valid sequence information (McMurdie and Holmes, 2014; Tedersoo et al., 2014, 2016). We applied a forward model selection procedure to identify significant drivers of fungal alpha diversity. Soil properties, topographic, and tree richness variables (excluding Shannon and Simpson diversity indices) were transformed by the natural logarithm. Coordinates of sampling locations were transformed into principal coordinates of neighborhood matrices (PCNM, Legendre et al., 2009) using the vegan package (Oksanen et al., 2013). The resulting vectors were incorporated into mixed effect models with the variable forest plot identity as a random factor. Correlations of the selected variables were inspected by variance inflation analysis (vif) carried out with the fmsb package using a threshold of 10. We applied a forward model selection procedure with linear mixed-effect models using the lme4 package (Bates et al., 2015) based on the Akaike Information Criterion corrected for small sample sizes (MuMIn package, Barton, 2018). The significance of model parameters was assessed by linear mixed effect models using the nlme package (Pinheiro et al., 2017) followed by Analysis of Variance (ANOVA) type II tests implemented in the car package (Fox and Weisberg, 2011). Shapiro–Wilk tests were applied to confirm that model residuals met normal distribution assumptions. The VarCorr function of the lme4 package was applied to derive the extent to which fungal richness variation was attributable to the random factor forest plot. Explained variance was partitioned to fixed effect factors using the hier.part package (Walsh and MacNally, 2013). The conditional and marginal coefficients of determination for the mixed effect models were calculated using the MuMIn package (Barton, 2018). The marginal coefficients of determination represent the amount of variance explained by the fixed factors while the conditional coefficients of determination indicate the amount of variance explained by both fixed and random factors (Nakagawa et al., 2013).

### Fungal Beta-Diversity Analysis

Beta-diversity values for the fungal and tree communities were calculated in terms of pairwise Bray–Curtis dissimilarities based on averaged (per tree species per plot) Hellinger-transformed abundance counts, representing percentage differences in community composition (Legendre and De Cáceres, 2013). Tree community abundance counts were based on the sampled trees and their eight neighboring tree individuals. Geographic distances between sampling locations, soil properties and topographic variables were standardized by natural logarithm transformation and averaged data were transformed to Euclidean distances.

To identify the environmental variables that explained the most fungal beta-diversity, multiple regressions of distance matrices were applied using the MRM function in the ecodist package (Goslee and Urban, 2007). The identity of forest plots was included as a fixed factor to account for the differences in pairwise sample comparisons within and between plots. Only variables showing significant correlation with fungal beta-diversity in partial Mantel tests after accounting for variations in geographical distances between sampling locations were considered for multiple regression analysis. Furthermore, variables were excluded if they had a variance inflation factor (vif) greater than 10, calculated using the vif function in the fmsb package (Nakazawa, 2014). Best subset model selection was carried out to identify the parameters that best explained fungal community turnover. The complete list of best model subsets is shown in **Supplementary Tables S4–S7**. The percentages of explained variance contributed by the remaining variables were calculated using the varpart function in the vegan package (Oksanen et al., 2013).

## Results

### Fungal Taxonomic Assignment

Pyrosequencing generated 1,155,299 raw sequences in total from the 394 soil samples collected. Clusters of least four of the 737,907 reads that passed the quality filtering were assigned to 5,665 fungal OTUs. Rarefaction curves for each fungal functional group investigated are available in **Supplementary Figure S2**. In total, 72, 56, and 49% of the fungal OTUs were classified at the order, family and genus levels, respectively.

We could assign 54% of the fungal OTUs to a functional group: 31% (1,768 OTUs), 7% (410 OTUs), 5% (320 OTUs), and 5% (310 OTUs) to saprotrophic, EcM, AM, and pathogenic fungi, respectively (**Supplementary Figure S3**). On the basis of the OTU numbers, saprotrophic fungi were predominantly Ascomycota (57%), followed by Basidiomycota (37%), Mucoromycota (5%) and Chytridiomycota (1%). Agaricales was the most diverse order of saprotrophic fungi, accounting for about 20% of the OTUs (**Supplementary Table S2**). Pathogenic fungi were predominantly plant pathogens (76%), followed by mycoparasites (11%) and animal parasites (11%). This group was strongly dominated by Ascomycota (87% of the OTUs), followed by Basidiomycota (10%) and Chytridiomycota (2%). The five most diverse orders of pathogenic fungi were the Capnodiales (26%), Pleosporales (14%), Hypocreales (12%), and Cantharellales (5%, **Supplementary Table S3**). AM fungi, which are monophyletic Glomeromycota, were clearly dominated by members of the order Glomerales (76% of the OTUs), followed by Archaeosporales (10%), Diversisporales (4%) and Paraglomerales (4%). EcM fungi were almost completely made up of Basidiomycota (86% of the OTUs) and Ascomycota (13%). The most diverse orders of these fungi were Agaricales (24%) and Thelephorales (22%).

### Effects of Spatial, Soil Property and Tree Diversity Variables on Soil Fungal Alpha Diversity

**FIGURE 2:**
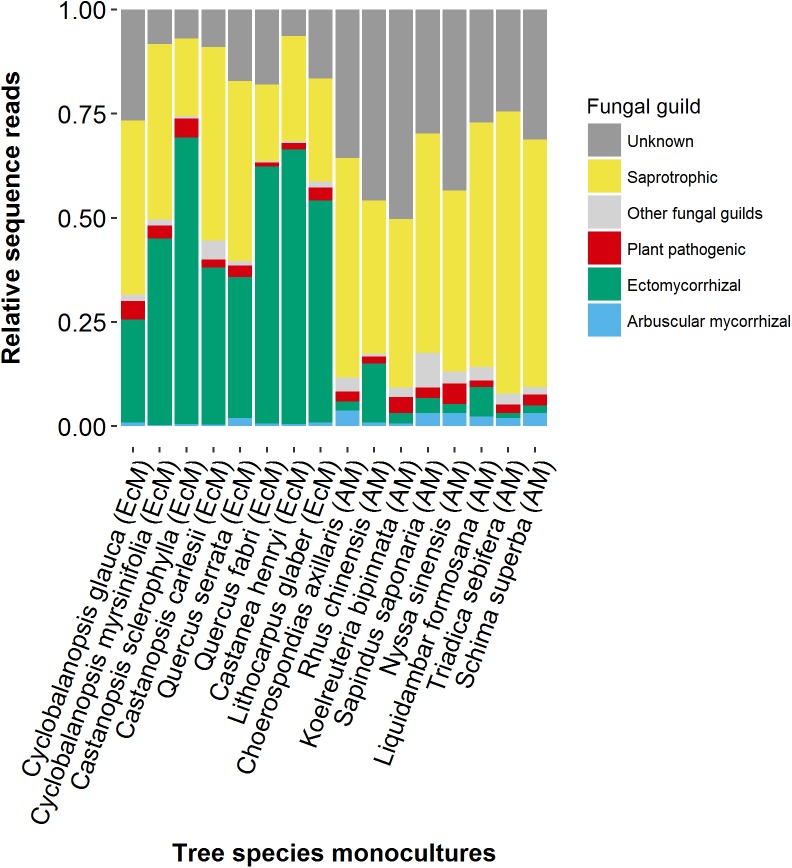
Relative abundances of fungal functional groups in tree monocultures. The mycorrhizal type (EcM or AM) of each tree species is indicated in brackets. Atypically high numbers of EcM fungal sequences were found in two and one of the five replicates of *Liquidambar formosana* and *Sapindus saponaria* rooting zone soil samples, respectively (10-fold higher than in the other replicates). These numbers are not displayed in this graphic, but they were included in the statistical analyses.

**FIGURE 3:**
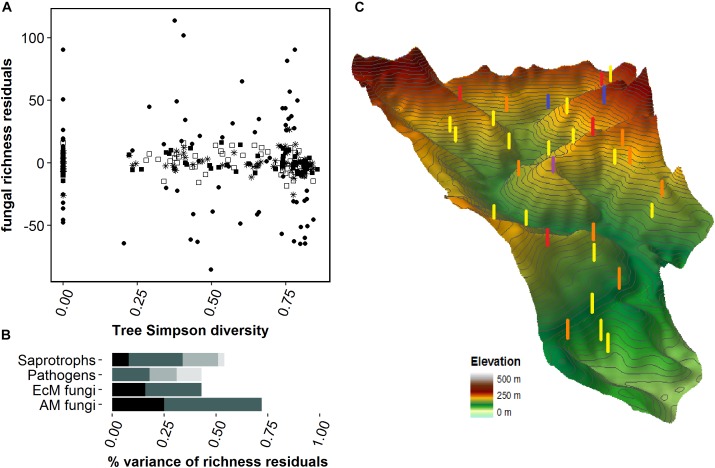
Correlation of fungal richness residuals with tree diversity **(A)**. The scatterplots show residuals of saprotrophic (closed circles), pathogenic (star), ectomycorrhizal (filled squares) and arbuscular mycorrhizal (open squares) fungal richness. Bar plots **(B)** show contributions of the following abiotic and spatial variables to variations in fungal richness, in decreasing intensity of shading: soil chemistry, geographical principal component neighborhood matrix eigenvectors, forest plot and the joint contribution of the last two factors. The three-dimensional elevation map **(C)** of the study area highlights the locations of forest plots, with color coding indicating the diversity levels of monocultures (yellow), two-species mixtures (orange), four-species mixtures (red), eight-species mixtures (blue), and 16-species mixture (purple).

Mean numbers of saprotrophic, pathogenic, AM and EcM fungal species per tree species within a forest plot were 78, 20, 14, and 9, respectively. The relative abundances of EcM fungal sequences were greater in the rooting zones of EcM-forming tree species than in those of AM-forming trees, especially in the tree monocultures (**Figure 2**). Linear regression analysis of fungal richness residuals and tree alpha diversity (Simpson indices) revealed no significant correlations (**Figure 3**). For the four fungal groups investigated, the final models selected by the forward selection procedure did not include any tree richness related variables (**Table 1**). In total, spatial variables and soil properties explained 75, 53, 46, and 44% of AM, saprotrophic, EcM and pathogenic fungal richness residual variance, respectively. Thus about 28–57% of the variation in fungal richness remained unexplained. Most of the explained variance of the fungal richness residuals was attributable to the PCNM spatial eigenvectors: 47, 27, 26, and 18% for AM, EcM, saprotrophic and pathogenic fungi, respectively. Of the soil properties tested, *N*_tot_ contributed significantly to the explained variation in saprotrophic fungal richness residuals (8%), soil water content to EcM fungal (16%) and soil water content, slope and effective cation exchange capacity to AM fungal richness residuals (25%). The residual richness of pathogenic fungi was correlated only with the spatial PCNM eigenvectors. The variable forest plot, which was included as a random factor in the linear mixed effect models, contributed strongly to the total amount of variance explained by the final models for saprotrophic (17%) and pathogenic fungi (13%). It did not affect the model strength for the mycorrhizal fungi richness residuals.

**Table 1:** Final model explaining fungal richness residuals in correlations of saprotrophic, pathogenic, ectomycorrhizal and arbuscular mycorrhizal fungi as functions of spatial, environmental and tree diversity variables.

Saprotrophic fungi				Pathogenic fungi				Ectomycorrhizal fungi				Arbuscular mycorrhizal fungi			
			
	Chisq	Df	*P*		Chisq	Df	*P*		Chisq	Df	*P*		Chisq	Df	*P*
***N*_tot_**	6.2	1	0.013	**PCNM36**	10.7	1	0.00	**SWC**	19.7	1	0.000	**PCNM25**	9.2	1	0.002
**PCNM8**	8.5	1	0.004	**PCNM24**	5.4	1	0.02	**PCNM5**	9.7	1	0.002	**PCNM13**	18.8	1	0.000
**PCNM21**	6.4	1	0.011	**PCNM40**	5.2	1	0.02	**PCNM12**	6.2	1	0.012	**PCNM34**	4.8	1	0.029
**PCNM3**	6.0	1	0.014					**PCNM1**	6.0	1	0.014	**SWC**	30.9	1	0.000
								**PCNM29**	5.5	1	0.019	**PCNM3**	29.5	1	0.000
***R*^2^_m_**	**0.36**			***R*^2^_m_**	**0.19**			**PCNM14**	4.1	1	0.043	**PCNM4**	15.4	1	0.000
***R*^2^_c_**	**0.53**			***R*^2^_c_**	**0.44**							**SLOPE**	24.0	1	0.000
								***R*^2^_m_**	**0.46**			**PCNM5**	10.0	1	0.002
								***R*^2^_c_**	**0.46**			**PCNM9**	9.4	1	0.002
												**CEC**	7.1	1	0.008
												**PCNM22**	7.1	1	0.008
												**PCNM35**	4.6	1	0.031
												**PCNM32**	4.4	1	0.036
															
												***R*^2^_m_**	**0.75**		
												***R*^2^_c_**	**0.75**		


*SWC, soil water content; CEC, effective cation exchange capacity; PCNM, Principal Component Neighborhood Matrices of geographical sampling locations. Linear mixed effect models include experimental forest plot as a random factor. Values reported are the marginal amounts of explained variance (R^*2*^_m_) attributed to the fixed variables only and the conditional amount of explained variance (R^*2*^_c_) attributed to the summed contributions of fixed and random factors (forest plot)*.

### Effects of Differences in Spatial, Soil Property and Tree Community Variables on Soil Fungal Community Structure

Partial Mantel tests, after accounting for differences in geographic distances between samples, showed that at least one of the tree community variables investigated was significantly correlated with differences in pathogenic, AM and EcM fungal community structure (**Table 2**). The correlation between tree and saprotrophic community composition was close to the Bonferroni-corrected α = 0.05 significance level. Differences in AM fungal and pathogen community structure were significantly correlated with tree community composition. EcM fungal community structure was significantly correlated with sample tree identity, sample tree mycorrhiza type (EcM vs. AM), EcM tree abundance and EcM tree richness.

**Table 2:** Partial Mantel correlations, after accounting for dissimilarities in geographic location, of fungal and environmental as well as tree community dissimilarities for the fungal functional groups indicated.

		Saprotrophic fungi		Pathogenic fungi		AM fungi		EcM fungi	
					
	Variable	*R*	*P*	*R*	*P*	*R*	*P*	*R*	*P*
(1)	Forest plot	0.21	0.0001	**0.16**	0.0001	**0.14**	0.0001	**0.16**	0.0001
(2)	Tree community composition	0.11	0.0024	**0.22**	0.0001	**0.15**	0.0003	0.07	0.0292
(3)	Tree species identity	0.01	0.1668	0.02	0.0738	0.03	0.0492	**0.13**	0.0001
(4)	Sample tree AM/EcM type	0.02	0.0677	0.00	0.306	0.03	0.0729	**0.32**	0.0001
(5)	Tree richness	0.00	0.8481	0.03	0.1732	0.01	0.3838	0.05	0.0901
(6)	Tree Shannon diversity	0.00	0.8736	0.03	0.1685	0.01	0.3561	0.05	0.0885
(7)	Tree Simpson diversity	0.00	0.8903	0.04	0.162	0.03	0.2673	0.03	0.2373
(8)	EcM tree abundance	0.00	0.5179	0.05	0.1991	0.00	0.7535	**0.21**	0.0004
(9)	AM tree abundance	0.00	0.5649	0.02	0.3418	0.08	0.0905	0.06	0.0863
(10)	EcM tree richness	0.00	0.7971	0.06	0.1652	0.00	0.9385	**0.19**	0.0003
(11)	AM tree richness	0.00	0.6219	0.00	0.4885	0.00	0.4695	0.00	0.1234
(12)	pH (H_2_0)	**0.38**	0.0001	0.20	0.0028	**0.41**	0.0001	0.12	0.0141
(13)	*N*_tot_	0.13	0.0214	0.06	0.1714	0.08	0.1225	0.07	0.0979
(14)	*C*_tot_	**0.22**	0.0001	**0.19**	0.0016	**0.26**	0.0004	0.09	0.0418
(15)	*C*:*N* ratio	**0.41**	0.0001	**0.31**	0.0001	**0.40**	0.0001	0.08	0.0497
(16)	BS	**0.35**	0.0001	0.19	0.0038	**0.35**	0.0001	0.04	0.2378
(17)	CEC	**0.23**	0.0001	0.07	0.0956	**0.26**	0.0002	0.11	0.0272
(18)	Soil water content	**0.18**	0.0005	0.18	0.0092	0.12	0.0368	0.09	0.0331
(19)	Altitude	0.00	0.96	0.00	0.8169	0.00	0.644	0.00	0.948
(20)	Slope	0.07	0.0798	0.00	0.4638	0.06	0.1671	0.00	0.6141


**CEC, effective cation exchange capacity; BS, base saturation; N_*tot*_, total nitrogen content; C_*tot*_, total carbon content; C/N ratio, total carbon to total nitrogen ratio. Dissimilarity matrices of all the given variables were used in the partial Mantel test analysis. The significant alpha level (<0.05) was Bonferroni-corrected based on the number of variables tested (α < 0.0023)*. Bolded values indicate variables that are significantly correlated.*

**FIGURE 4:**
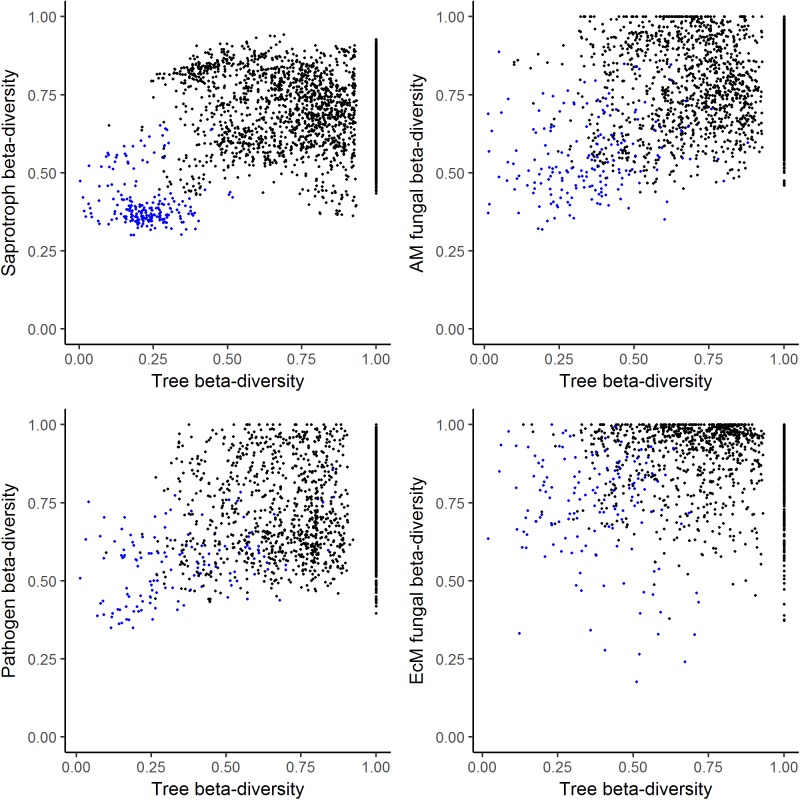
Relationship between fungal and tree beta-diversity (expressed as pairwise Bray–Curtis dissimilarities). Black circles show results of across forest plot comparisons and blue circles results of within forest plot comparisons (between differing tree species).

Scatterplots of pairwise Bray–Curtis dissimilarities showing correlations between tree beta-diversity and that of the specific fungal groups differed visibly (**Figure 4**). Saprotrophic fungal communities showed the least community turnover of all fungal groups, partly overlapping throughout the study site as the dissimilarity value for all pairwise community comparisons was <1 (which would represent 100% community dissimilarity). Community turnover within forest plots was much smaller than that between forest plots. There was no detectable trend in saprotroph community turnover associated with tree community composition. AM and pathogen fungal communities showed less pronounced separation of within- and between-forest plot community comparisons, implying some correlation with tree community structure. Strongly differing fungal communities were detected in some comparisons of soil samples from plots with ≥30% differences in tree community composition. EcM fungi formed very distinct communities and many pairwise sample comparisons showed no overlap of fungal species. EcM fungal communities showed the highest pairwise community dissimilarities of the four functional groups, with a mean Bray–Curtis dissimilarity of 0.89, followed by 0.78, 0.74, and 0.69 for AM, pathogenic and saprotrophic fungal communities, respectively. However, EcM fungal communities also showed the highest overlap of two sampled communities of a fungal functional group (approximately 80% EcM fungal community similarity). Tree communities with differences in composition as low as 13% had non-overlapping EcM fungal communities. There were no indications of any correlation between EcM fungal and tree community composition.

Following partial Mantel test analysis we selected the best model subsets for identifying the parameters that best explained differences in fungal community structure (**Table 3**). Abiotic soil properties explained the most variance in saprotrophic, pathogenic and AM community composition. The relevant abiotic variables for saprotroph community turnover were soil total carbon amount, carbon to nitrogen ratio, effective cation exchange capacity, base saturation and soil water content. Carbon to nitrogen ratio was also included in the final models of pathogenic and AM fungal community structure; in addition the latter model contained the abiotic soil properties pH and effective cation exchange capacity. The influence of tree community variables on fungal species turnover was strongly dependent on the fungal functional group investigated. Collectively, the tree-related variables that were found to be significant in the partial Mantel tests explained 11% of differences in EcM fungal community structure in the final MRM models. Although significant correlations were detected between tree community dissimilarity and both pathogenic and AM fungal community structure, the percentages of variance these correlations explained were very low: 5 and 2%, respectively. Spatial variables explained a large part of saprotroph and EcM fungal community variation but were only negligibly correlated with pathogenic and AM fungal community composition. Thus all of the three variable classes, biotic, abiotic and spatial variables, showed important correlations with soil fungal community structure, but the extent to which they did so greatly varied among the functional fungal groups. The best model subsets explained 54, 43, 26, and 23% of differences in saprotrophic, AM, EcM and pathogenic fungal community structure, respectively, leaving a large part of fungal community variation unexplained.

**Table 3:** Best subsets of environmental dissimilarity models explaining fungal beta-diversity.

	Saprotrophic fungi		Pathogenic fungi		Arbuscular mycorrhizal fungi		Ectomycorrhizal fungi	
				
	Model	Variance	Model	Variance	Model	Variance	Model	Variance
	Plot location	16%	Location	2%	Location	3%	Plot location	14%
			Tree comp	5%	Tree comp	2%	Sample tree ID	11%
							Sample tree	
							AM/EcM type	
							EcM tree abundance^∗^	
	*C*_tot_	19%	CN	9%	pH	22%		
	CN				CN			
	CEC				CEC			
	BS							
	SWC							
***R*^2^_,_*P***	**54%**	**0.0001**	**23%**	**0.0001**	**43%**	**0.0001**	**26%**	**0.0001**


*Environmental predictors are those that were retained in the best subsets of multiple regression models. All environmental variables and species information were transformed to dissimilarity matrices (see section “Materials and Methods”). CEC, effective cation exchange capacity; BS, base saturation; SWC, soil water content; Tree comp, tree community composition; variance, specific contributions of summarized soil variables, topography and tree composition to the total amount of variance. ^∗^EcM tree abundance could be replaced by the variable EcM tree richness, which showed very similar model performance.*

## Discussion

Soil fungi are a diverse (Blackwell, 2011; Taylor et al., 2014) and very heterogeneous group of organisms (Nguyen et al., 2016a). Our unprecedented study comprised the systematic analysis of four main fungal functional subgroups: saprotrophic, plant pathogenic, AM and EcM fungi. The subtropics constitute a zone of transition from temperate forests dominated by ectomycorrhizal symbiosis to tropical forests dominated by arbuscular mycorrhizal symbiosis. The subtropics thus harbor a high diversity of evergreen and deciduous tree species, and the number of AM and EcM forming trees occurring there is balanced, enabling the investigation of a broad range of fungal functional groups (Toju et al., 2014). Furthermore, the availability of a large-scale experimental assembly of native subtropical tree species enabled the quantification of the independent individual contributions made by tree community structure and soil properties and topography to soil fungal assembly. In contrast, tree community composition and richness (biotic variables) and soil properties (abiotic variables) are intermingled in naturally evolved forests. Our results provide evidence for a highly differentiated pattern in fungal-tree and fungal-environment (abiotic) relationships for all the fungal groups investigated. As expected, EcM fungal community assembly showed the strongest correlation with tree community variables, while saprotroph community assembly was driven only by abiotic spatial variables and soil properties. Against our expectations, AM fungal and tree community structure were significantly but weakly related. Fungal richness was not correlated with any of the tree community variables assessed. The strong influence of spatial variables and abiotic soil properties on fungal community assembly implies considerable early-stage environmental filtering and dispersal limitation.

### Spatial Variables and Abiotic Soil Properties Contributed to Variation in Fungal Richness

It was predominantly spatial variables, but also soil properties, that influenced fungal richness. The strong spatial effects might result from variations in recent fungal spore inputs, and unknown legacy effects of the vegetation previously at the site may also contribute. Such effects would be extremely difficult to quantify. The experimental site was directly surrounded by forest plantations to the north, west and east. Inputs from these forests would depend on multiple factors, including their composition, maturity and climatic factors. Similarly, the two tree species that were dominant in the clear-cut forest plantation are known—*Cunninghamia lanceolata* (AM) and *Pinus massoniana* (EcM)—but they were randomly distributed and the exact previous positions of these species and other minor components of the previous stands were not recorded.

Abiotic soil properties moderately impacted the variation in fungal richness of saprotrophic (8%, total nitrogen content), EcM (16%, soil water content) and AM fungi (25%, soil water content, slope, effective cation exchange capacity). The significant effect of soil nitrogen on saprotroph richness could be explained by the major limitation of this soil resource in our forests, where plant-microbial competition for soil nitrogen was reported previously (Pei et al., 2016). AM fungi depend on carbon provided by their host plants. The link between AM fungal richness and the two abiotic variables slope and effective cation exchange capacity could be related to this dependency. At our experimental site, slope was one of the main predictors of soil fertility (Scholten et al., 2017) which might impact tree productivity, thereby influencing the amount of rhizodeposition by host plants. Cation exchange capacity can be attributed mainly to soil aluminum content and aluminum negatively affects tree height (Scholten et al., 2017). Aluminum stress has been reported to hamper fine root growth and nutrient acquisition by trees (Marschner, 1991; Kinraide, 2008; de Wit et al., 2010). The detectable decrease in tree fitness due to metal toxicity might have led to fewer resources being translocated to the mycorrhizal fungal partner and fewer colonization sites due to negative impacts on root structure. None of the topographic variables and abiotic soil properties analyzed correlated with plant pathogen fungal richness. This could be due to the primary dependency of the pathogenic fungi on the living plant tissue. However, many pathogens spend their lifecycle partly as saprotrophs and thus a correlation with abiotic soil properties would have been expected (Kabbage et al., 2015).

None of the fungal groups analyzed, saprotrophs, pathogens, AM or EcM fungi, showed significant correlations between species richness and tree diversity. Similarly, no causal relationship of fungal richness and richness of fungal functional groups with plant diversity was found in a global study by Tedersoo et al. (2014). Only ectomycorrhizal fungal richness was globally correlated with the relative proportion and richness of EcM plants. In addition, in a regional study, Tedersoo et al. (2016) concluded that soil resources and tree species identity have greater effects on the diversity of soil biota than tree species richness *per se*. This is also supported by the observational study of Scheibe et al. (2015) conducted in German temperate broadleaved forests. Specific tree fungal richness relationships were found by Liang et al. (2015) and Nguyen et al. (2016b). Nguyen et al. (2016b) reported, from the American IDENT experimental site, a correlation between EcM fungal richness and plant phylogenetic diversity which was caused by the host specific EcM fungal species associated with gymno- and angiosperms. Several local and regional observational studies have reported strong tree species effects (Urbanova et al., 2015) and a correlation between plant and fungal richness (Gao et al., 2013; Martínez-García et al., 2015). Liang et al. (2015) found a negative relationship between AM fungi and tree diversity in subtropical restoration sites, which was attributed to a (presumably) higher carbon flux to the belowground compartment in less diverse and fast-growing forests compared to diverse but light-limited secondary forests.

We determined fungal richness based on the presence and absence of diagnostic sequences in DNA extracted from bulk soil samples. However, tree community effects might first be discernible in changes in fungal abundances, before fungal species disappear from the detectable soil DNA pool. Fungal species could be detectable for several years through DNA content extracted from inactive spores, dead mycelium and extracellular DNA (Levy-Booth et al., 2007; Nielsen et al., 2007) even when they are not actually living under present-day conditions. Furthermore, plants must be successfully colonized by fungi before differences in their fungal symbionts’ efficiency can have any effect (Dickie et al., 2015), so relatively inefficient fungi may reside in habitats spanning fairly wide ranges of environmental conditions for considerable periods. In grassland experiments, time lags of several years were reported before changes in the plant community composition led to detectable changes in the composition of the soil microbial community (Eisenhauer et al., 2010). Thus the effects of tree species identity and tree species richness on fungal richness could still become detectable in future years of forest development. In comparison, the experimental forests in the study of Tedersoo et al. (2016) were well-grown with a closed canopy.

### Differences in Tree Community Variables Significantly Affected Community Structure for Fungal Groups Other Than Saprotrophs

Consistent with their dominant influence on fungal richness, spatial variables and abiotic soil properties also had the strongest effects on fungal community assembly. These variables explained a large proportion of saprotroph beta-diversity without there being any effect of tree related variables. At the time of the study, tree saplings (including many evergreen tree species) provided only limited belowground carbon input through rhizodeposits and leaf litter. As saprotrophic fungi depend on dead rather than living plant tissue, this community was likely sustained by the carbon stock residing in stumps and dead roots from the previous forest plantation. Several other studies have also revealed a strong influence of abiotic conditions on saprotroph and whole fungal communities (Wu et al., 2013; Prévost-Bouré et al., 2014; Tedersoo et al., 2014; Pei et al., 2016) while significant impacts of tree species community composition on saprotroph community structure have been found as well (Nguyen et al., 2016b; Schappe et al., 2017). This divergence in results regarding the impact of spatial, biotic and abiotic drivers on soil fungal community composition is also evident from studies focusing on the AM and EcM fungal subgroups (Öpik and Peay, 2016). Many AM fungi are distributed globally (Davison et al., 2015) with global AM fungal diversity (about 300 described to 3000 estimated species, Krüger et al., 2011; Buscot, 2015) being extremely low compared to that of AM host plants (several hundreds of thousands, Wang and Qiu, 2006). The AM fungi have therefore long been thought to be host generalists. Many studies report strong environmental filtering of AM fungal communities by soil properties such as pH (Dumbrell et al., 2010), distance and CN (Dumbrell et al., 2010; Davison et al., 2016), soil texture and soil moisture (Freitas et al., 2014), and temperature and soil P (Davison et al., 2016). The AM fungal communities in our young subtropical forests were strongly structured by abiotic (pH, CEC, and CN) and spatial variables. However, several studies found that host plant identity has effects on AM community assembly (Öpik et al., 2009; Wubet et al., 2009; Martínez-García et al., 2015) and it has been suggested that discrete regional and habitat specific fungal pools exist, indicating context dependent host specificity (Öpik and Peay, 2016). We found a significant but weak effect of tree community composition on AM fungal community structure. It should be noted that a rich herb layer, dominated in terms of biomass by ferns, developed at our experimental forest site in addition to the tree saplings planted there (Germany et al., 2017). In Southwest China, Zhang et al. (2004) found that the majority of the fern species they investigated were AM hosts. Substantial amounts of fern-associated AM fungi presumably persisted in the soil and could have impacted the tree-associated AM fungal community composition that we identified and potential relationships with tree variable effects.

The evolutionary development of EcM fungi from white and brown rot fungi took place convergently multiple times during the past 125 million years, reaching an EcM fungal species diversity approximately equal to that of EcM host plants (about 6000, Buscot, 2015). Thus EcM fungi have been assumed to be specific in nature (Öpik and Peay, 2016) and many studies, including our results, support a strong host effect (Ishida et al., 2007; Tedersoo et al., 2008, 2010; Ding et al., 2011). The linkage between tree and EcM communities, together with the high diversity of EcM fungi found, presumably reflects an early stage in the establishment of a complete EcM fungal community at our experimental site. However, the true host preference of EcM fungi may rely causally on the specific environmental conditions created by the host (Öpik and Peay, 2016), since strong environmental drivers of EcM community composition have been reported previously (Huang et al., 2014; Glassman et al., 2017; van der Linde et al., 2018), to the extent that EcM species can be indicators for key environmental variables (van der Linde et al., 2018). At our site, spatial and tree related variables structured EcM community composition while abiotic soil properties did not. Pathogenic fungal community composition was related to tree community structure, spatial and abiotic variables (CN content). The simultaneous lack of host effects (as indicated by a lack of correlation with differences in tree species identity) could indicate that local tree diversity and non-host neighboring tree species have played prominent roles. A similar pattern was found for biotrophic foliar pathogens in a young temperate experimental forest (Hantsch et al., 2014). Hantsch et al. (2014) concluded that particular non-host species (fast growing conifers in their study) in the vicinity of a target tree species (*Tilia cordata* and *Quercus petraea*) may impede or facilitate fungal pathogen infection depending on the identity of the species and its proportion in the local neighborhood.

A large proportion of the variation in fungal community structure and richness remained unexplained by the variables that we studied. There are numerous possible reasons for this finding (Bahram et al., 2015). Some significant effects may have been missed, because influential environmental variables were not measured. For example, Tedersoo et al. (2016) found herb cover and tree basal area to be strongly associated with fungal richness. These variables were not quantified within the framework of our study. In addition, manganese was present in high concentrations, and this has been reported to have a negative influence on tree height (Scholten et al., 2017) and potentially also to have a negative impact on EcM fungal diversity (Huang et al., 2014). However, stochastic processes may also have major effects on fungal community assembly (Powell et al., 2015; Bahram et al., 2016). Furthermore, our sequence-based data on fungal community composition may be insufficiently precise and representative, and this would certainly account for most of the unexplained variation.

## Conclusion

We quantitatively assessed the independent contributions made by spatial, abiotic (soil properties and topography) and biotic (tree community structure) variables to soil fungal community structure in a study facilitated by the experimental set-up of the tree diversity forest plots that we investigated. Our results suggest that strong environmental filtering and dispersal limitation were the most important drivers of fungal community assembly in young subtropical forests. The influence of biotic tree community variables could already be detected in mycorrhizal and pathogen fungal groups. Due to the limited size of the tree saplings and thus of the carbon input to the ecosystem by rhizodeposits and leaf litter, we expect there to be increasingly strong tree related effects on fungal community composition as forest development proceeds. Despite focusing on an early stage of forest development, our study clearly indicates that different functional groups of soil fungi respond specifically to different soil and vegetation variables, and that these specific responses may be at either the species richness or the community composition level. Ongoing studies on context-dependent community assembly of soil fungi should therefore take into account functional guilds within the fungi.

## Data Availability

Relevant materials and protocols will be made available upon request. Datasets of the raw sequences generated for this study can be found in the European Nucleotide Archive (https://www.ebi.ac.uk/ena/data/view/PRJEB12020) (Weißbecker et al. 2016). The bioinformatically processed sequence dataset and metadata can be found in the Zenodo repository (https://zenodo.org/record/1215505) (Weißbecker et al. 2018), as can the R scripts generated for the statistical analyses (https://zenodo.org/record/1401839). (Weißbecker and Wubet, 2018)

## Author Contributions

CW, FB, TW, and HB designed the study. CW performed the soil sampling and sample preparation. TS and PK provided soil measurements. GL, TW, and CW performed the bioinformatics analysis. CW and TW did the statistical analyses. CW, TW, FB, and HB outlined the manuscript, the first draft was written by CW. All authors contributed to revisions.

## Conflict of Interest Statement

The authors declare that the research was conducted in the absence of any commercial or financial relationships that could be construed as a potential conflict of interest.

## Footnotes

1 http://www.apsnet.org
