# Supplementary material for: Experimental Evidence of Functional Group-Dependent Effects of Tree Diversity on Soil Fungi in Subtropical Forests
Source: Front Microbiol. 2018 Oct 9;9:2312. doi: 10.3389/fmicb.2018.02312 (PMC6189305; doi:10.3389/fmicb.2018.02312)
Supplement: Supplementary file 1 [file Data_Sheet_1.DOCX]

Supplementary Material

Experimental evidence of functional group-dependent effects of tree diversity on soil fungi in subtropical forests

Christina Weißbecker*, Tesfaye Wubet, Guillaume Lentendu_,_ Peter Kühn, Thomas Scholten, Helge Bruelheide, François Buscot

*** Correspondence:** Christina Weißbecker: christina.weissbecker@ufz.de

## Supplementary Figures

v

1

2

4

8

16

|  | **Tree species** | **Type** | **No.** |  | **Tree species** | **Type** | **No.** |
| --- | --- | --- | --- | --- | --- | --- | --- |
|  | *Castanea henryi* (Skan) Rehd. & Wils. | EcM | 5 x 5 | v | *Cyclobalanopsis glauca* (Thunb.) Oerst. | EcM | 5 x 5 |
|  | *Nyssa sinensis* Oliver | AM | 5 x 5 |  | *Quercus fabri* Hance | EcM | 5 x 5 |
|  | *Liquidambar formosana* Hance | AM | 5 x 5 |  | *Rhus chinensis* Mill. | AM | 5 x 5 |
|  | *Sapindus saponaria* Linn. | AM | 5 x 5 |  | *Schima superba* Gardner & Champion | AM | 5 x 5 |
|  | *Choerospondias axillaris* (Roxb.) Burtt & Hill | AM | 5 x 5 |  | *Castanopsis eyrei* (Champ.) Tutcher/ *C. carlesii* (Hemsl.) Hay. | EcM | 5 x 5 |
|  | *Triadica sebifera* (L.) Small | AM | 5 x 5 |  | *Cyclobalanopsis myrsinifolia* (Blume) Oerst. | EcM | 5 x 5 |
|  | *Quercus serrata* Murray | EcM | 5 x 5 |  | *Lithocarpus glaber* (Thunb.) Nakai | AM | 5 x 5 |
|  | *Castanopsis sclerophylla* (Lindl.) Schott. | EcM | 5 x 5 |  | *Koelreuteria bipinnata* Franch. | AM | 5 x 5 |
| **Number of soil samples** | |  | **200** | **Number of soil samples** | |  | **200** |

**Supplementary Figure 1.** Broken-stick-design of the experimental forest plots. Plot design presented for the Biodiversity and Ecosystem Functioning (BEF) experiment China, study site A. The 16 species mix was sub divided in two times eight species mixtures. These were likewise partitioned in four, two and one tree species communities. Tree species are shown as symbols. Similarity of symbols was only chosen to emphasize the experimental design and does not imply any similarities or dissimilarities of tree species. No.: Number of samples.

**
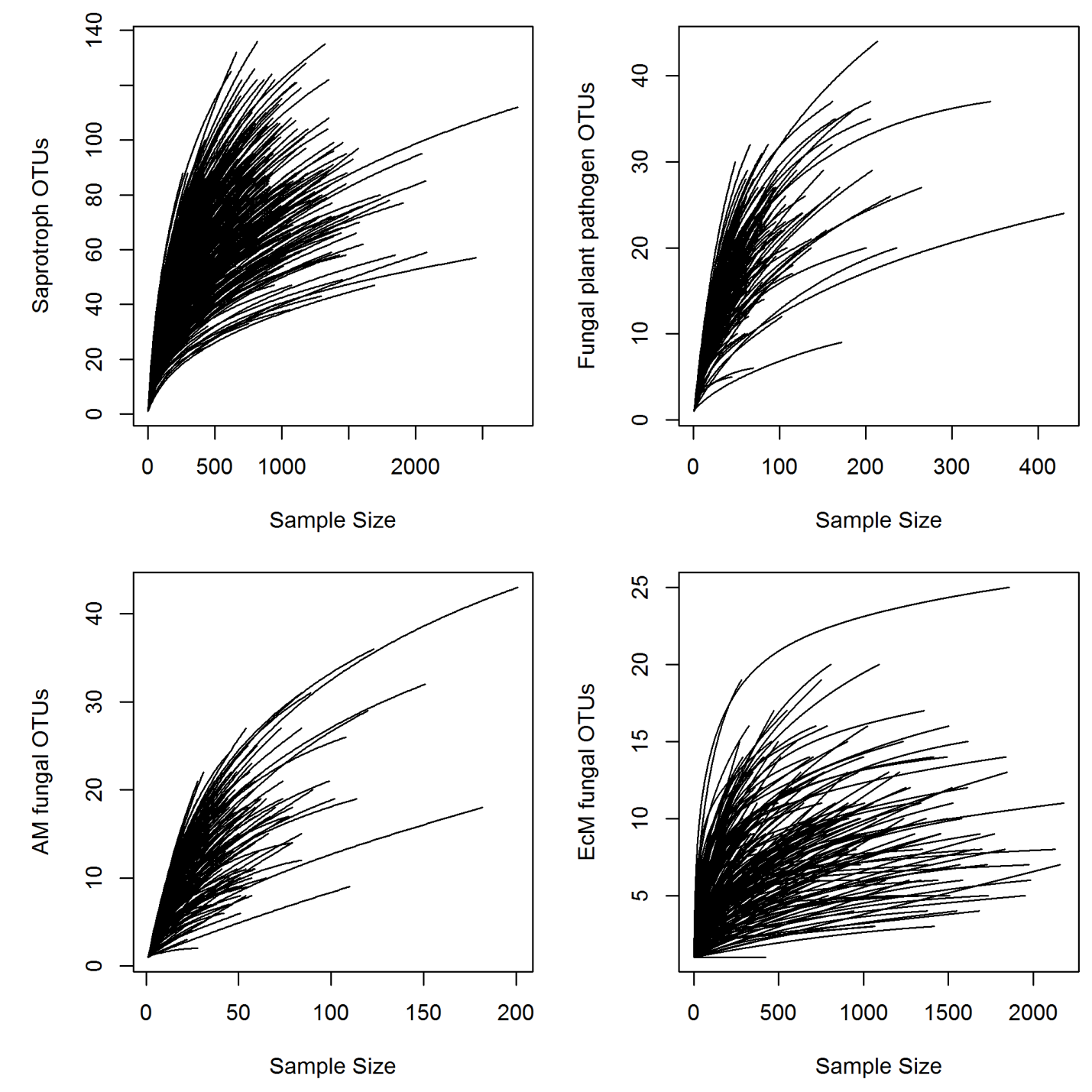
**

**Supplementary Figure 2.** Rarefaction curves for the main fungal functional groups after sequence quality filtering and removal of singleton, doubleton and tripleton operational taxonomic units (OTUs).


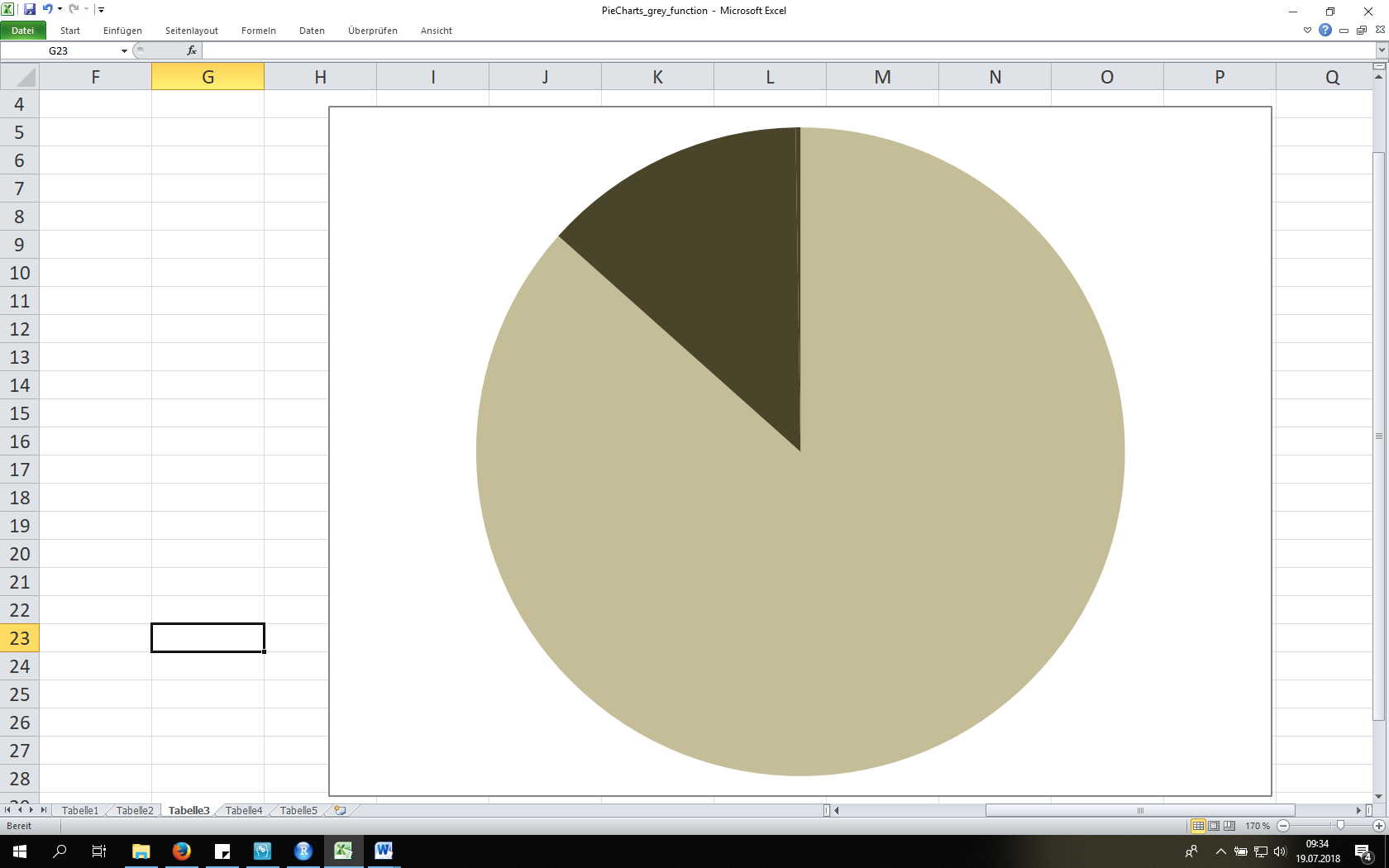

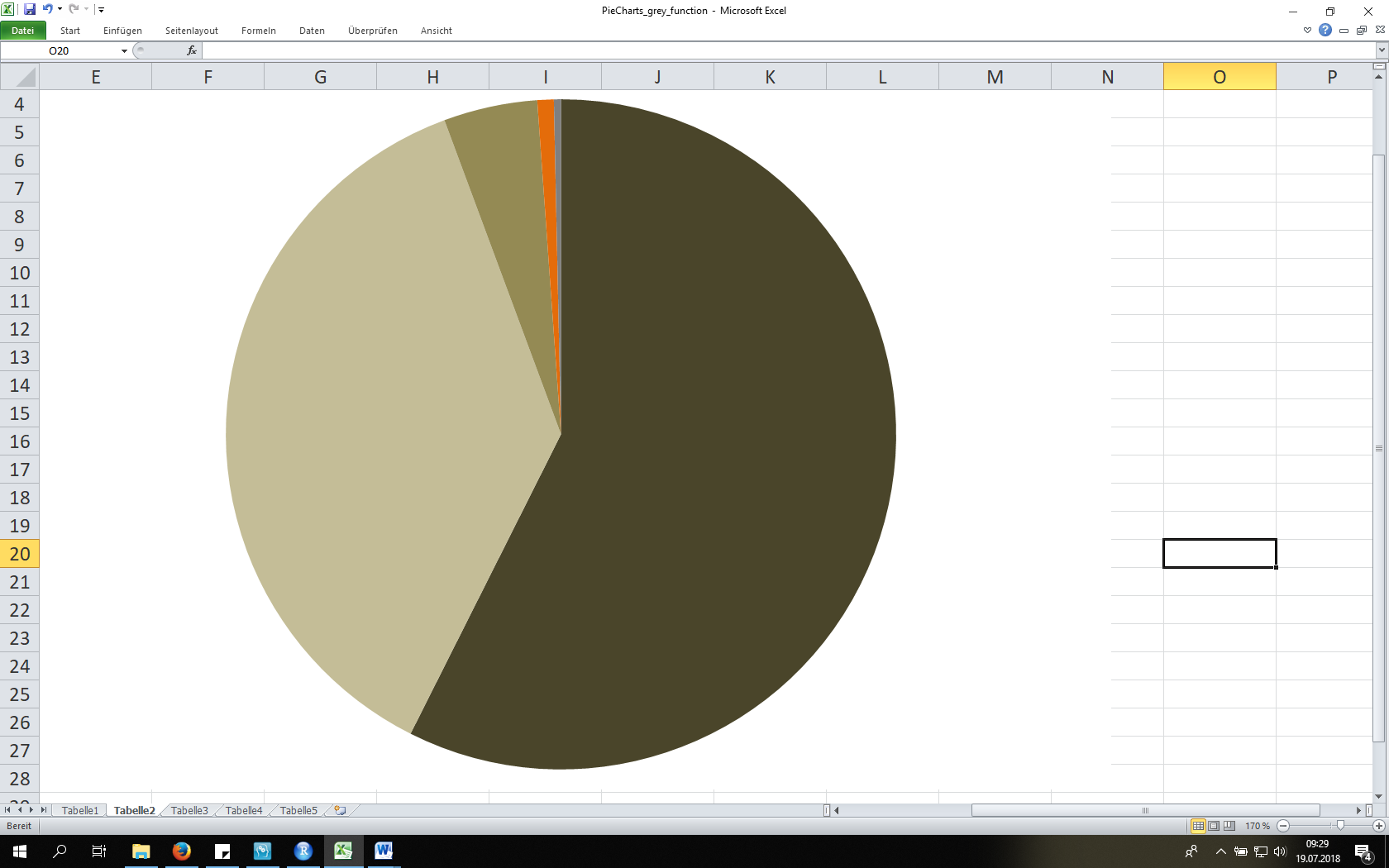

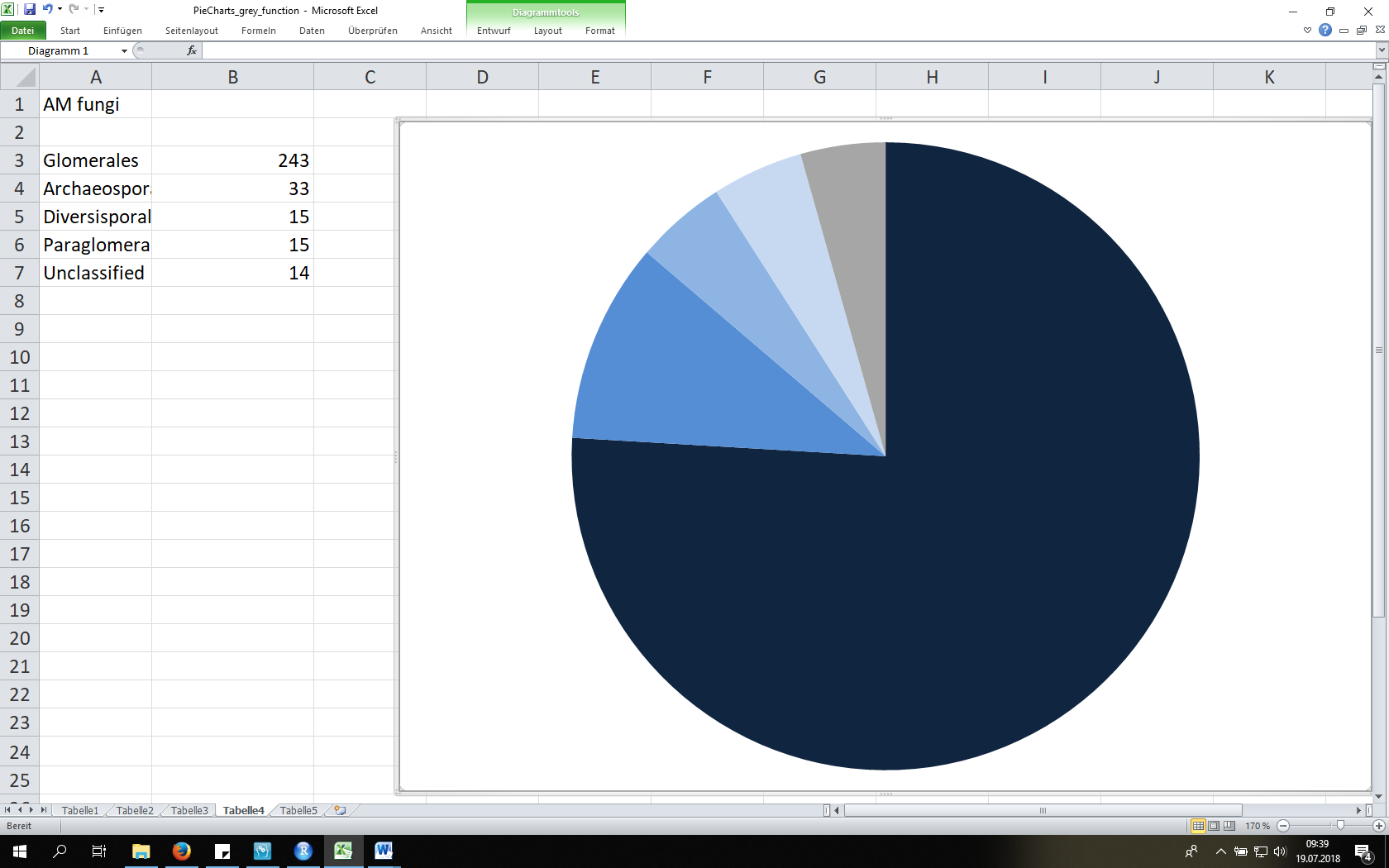


Asco-

mycota

Glomerales

Basidiomycota

Basidio-

mycota

Ascomycota

Archaeosporales

Diversisporales

Paraglomerales

Unclassified

Basidiomycota

Chytridiomycota

Entorrhizomycota

Mucoro-mycota

Chytridiomycota

Zoopago-mycota


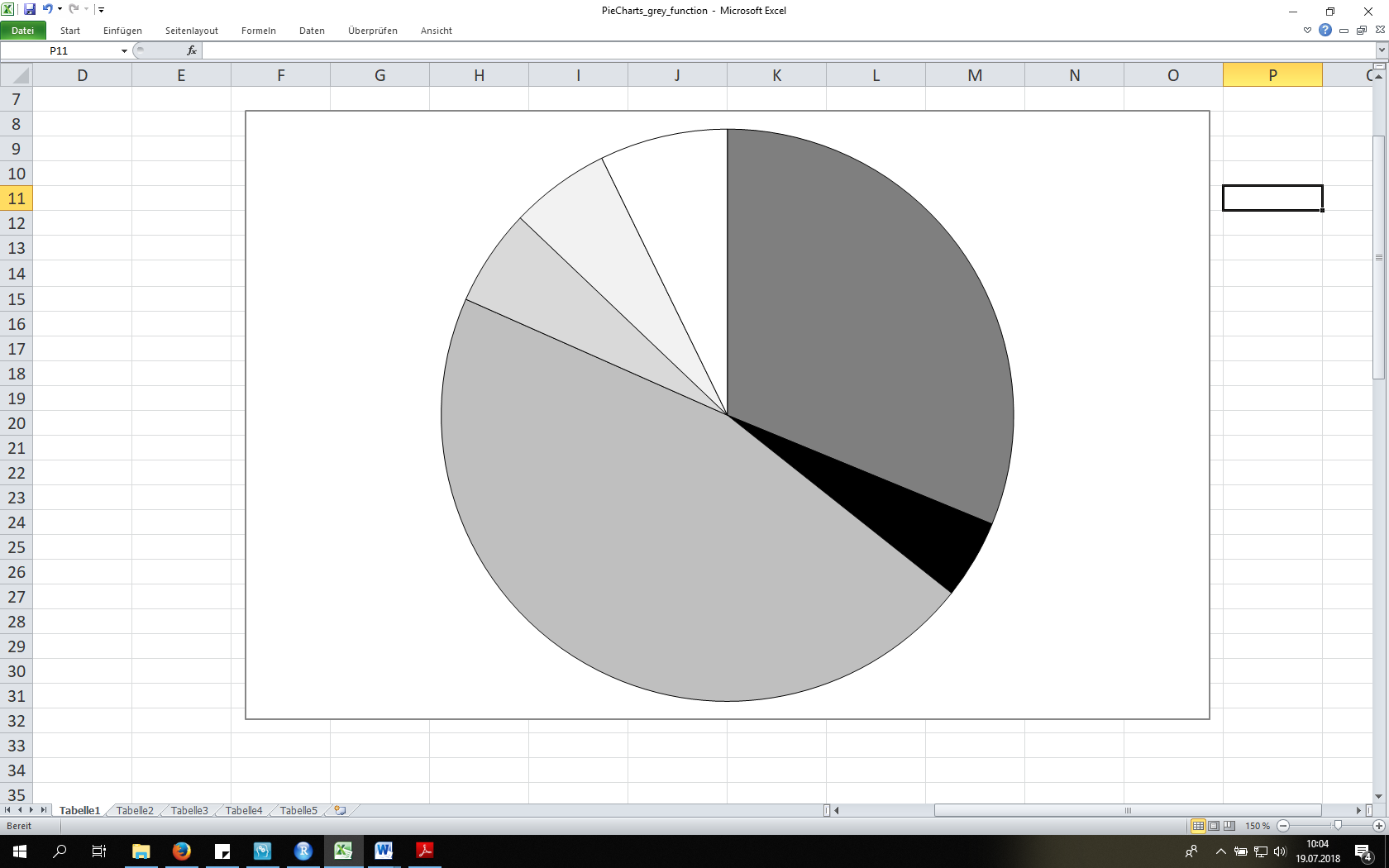

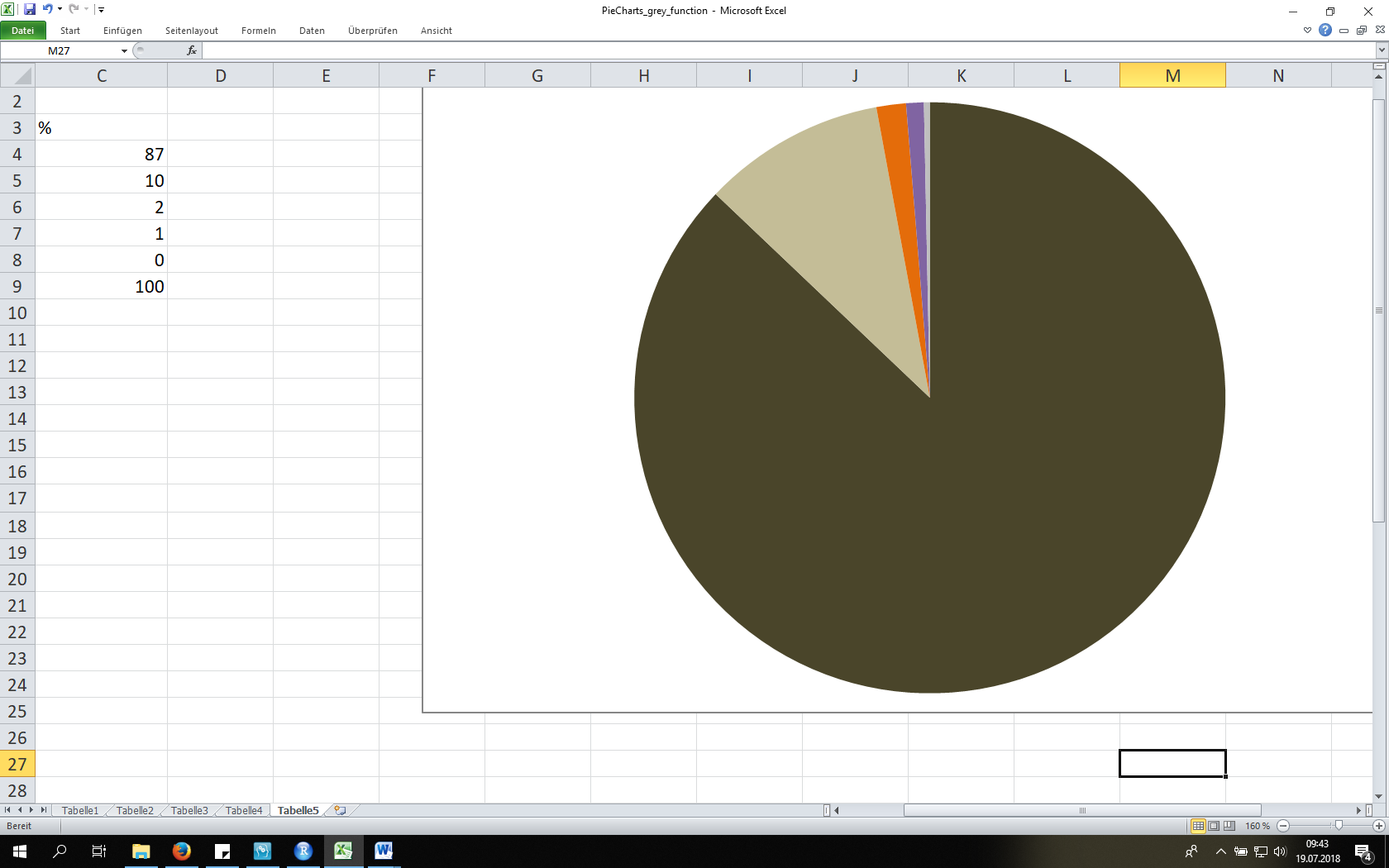


Saprotrophs

Ascomycota

Unknown

AM fungi

Pathogen

EcM fungi

Others

**Supplementary Figure 3.** Taxonomic composition of the main fungal functional groups based on operational taxonomic unit (OTU) counts. Taxonomic composition of saprotrophic, pathogenic and ectomycorrhiza fungi is given by phyla while it is presented by orders for arbuscular mycorrhiza fungi.

## Supplementary Tables

## Supplementary Table 1. Frequencies of the actual number of soil samples replicates included in the statistical sampling units.

| **Number of replicates included in a statistical sampling unit** | Number of sampling units containing the sequence information of the respective number of sample replicates | | | |
| --- | --- | --- | --- | --- |
|  | **Saprotrophic fungi** | **Plant pathogenic fungi** | **Ectomycorrhizal fungi** | **Arbuscular mycorrhizal fungi** |
| **1** | - | 15 | 17 | 13 |
| **2** | 2 | 17 | 6 | 16 |
| **3** | 10 | 18 | 6 | 15 |
| **4** | 15 | 11 | 14 | 11 |
| **5** | 53 | 6 | 17 | 16 |

## Supplementary Table 2. Taxonomic phyla and orders of saprotrophic soil fungi detected.

|  | **Saprotrophic fungi** | **OTU count** |  |  |  | **Saprotrophic fungi** | **OTU count** |
| --- | --- | --- | --- | --- | --- | --- | --- |
| **1** | Agaricales | 378 |  |  | **38** | Dothideales | 5 |
| **2** | Eurotiales | 143 |  |  | **39** | Myrmecridiales | 5 |
| **3** | Hypocreales | 139 |  |  | **40** | Ostropales | 5 |
| **4** | Archaeorhizomycetales | 127 |  |  | **41** | Phallales | 4 |
| **5** | Pleosporales | 99 |  |  | **42** | Tritirachiales | 4 |
| **6** | Helotiales | 81 |  |  | **43** | Tubeufiales | 4 |
| **7** | Tremellales | 78 |  |  | **44** | Onygenales | 4 |
| **8** | Mortierellales | 75 |  |  | **45** | Trichosporonales | 4 |
| **9** | Chaetothyriales | 71 |  |  | **46** | Agaricostilbales | 4 |
| **10** | Sordariales | 69 |  |  | **47** | Boliniales | 3 |
| **11** | Xylariales | 45 |  |  | **48** | Sporidiobolales | 3 |
| **12** | Trechisporales | 40 |  |  | **49** | Annulatascales | 2 |
| **13** | Geminibasidiales | 39 |  |  | **50** | Atractiellales | 2 |
| **14** | Orbiliales | 26 |  |  | **51** | Chytridiales | 2 |
| **15** | Saccharomycetales | 24 |  |  | **52** | Filobasidiales | 1 |
| **16** | Venturiales | 24 |  |  | **53** | Hysteriales | 1 |
| **17** | Auriculariales | 24 |  |  | **54** | Kriegeriales | 1 |
| **18** | Corticiales | 21 |  |  | **55** | Lichenostigmatales | 1 |
| **19** | Polyporales | 20 |  |  | **56** | Magnaporthales | 1 |
| **20** | Chaetosphaeriales | 18 |  |  | **57** | Pleurotheciales | 1 |
| **21** | Coniochaetales | 16 |  |  | **58** | Pyrenulales | 1 |
| **22** | Geoglossales | 14 |  |  | **59** | Rhizophydiales | 1 |
| **23** | GS31 | 14 |  |  | **60** | Chytridiales | 1 |
| **24** | Geastrales | 14 |  |  | **61** | Filobasidiales | 1 |
| **25** | Cantharellales | 12 |  |  |  |  |  |
| **26** | Capnodiales | 11 |  |  |  |  |  |
| **27** | Russulales | 10 |  |  |  |  |  |
| **28** | Umbelopsidales | 10 |  |  |  |  |  |
| **29** | Conioscyphales | 9 |  |  |  |  |  |
| **30** | Microascales | 8 |  |  |  | Ascomycota | 1015 |
| **31** | Spizellomycetales | 8 |  |  |  | Basidiomycota | 653 |
| **32** | Boletales | 8 |  |  |  | Mucoromycota | 80 |
| **33** | Dacrymycetales | 7 |  |  |  | Chytridiomycota | 14 |
| **34** | Hymenochaetales | 6 |  |  |  | Zoopagomycota | 6 |
| **35** | Kickxellales | 6 |  |  |  |  |  |
| **36** | Ophiostomatales | 6 |  |  |  |  |  |
| **37** | Pezizales | 6 |  |  |  |  |  |

## Supplementary Table 3. Taxonomic phyla and orders of pathogenic fungi, arbuscular mycorrhizal (AM) fungi and ectomycorrhizal fungi (EcM).

|  | **Pathogenic fungi** | **OTU number** |  |  |  | **AM Fungi** | **OTU numbers** |
| --- | --- | --- | --- | --- | --- | --- | --- |
| **1** | Capnodiales | 80 |  |  | **1** | Glomerales | 243 |
| **2** | Pleosporales | 43 |  |  | **2** | Archaeosporales | 33 |
| **3** | Hypocreales | 36 |  |  | **3** | Diversisporales | 15 |
| **4** | NA | 34 |  |  | **4** | Paraglomerales | 15 |
| **5** | Cantharellales | 16 |  |  |  |  |  |
| **6** | Glomerellales | 14 |  |  |  | Glomeromycota | 320 |
| **7** | Xylariales | 14 |  |  |  |  |  |
| **8** | Helotiales | 13 |  |  |  |  |  |
| **9** | Magnaporthales | 9 |  |  |  | **EcM Fungi** | **OTU numbers** |
| **10** | Diaporthales | 8 |  |  | **1** | Agaricales | 98 |
| **11** | Botryosphaeriales | 7 |  |  | **2** | Thelephorales | 89 |
| **12** | Togniniales | 6 |  |  | **3** | Sebacinales | 61 |
| **13** | Chaetothyriales | 5 |  |  | **4** | Russulales | 33 |
| **14** | Polyporales | 5 |  |  | **5** | Cantharellales | 31 |
| **15** | Entorrhizales | 3 |  |  | **6** | Helotiales | 23 |
| **16** | Platygloeales | 3 |  |  | **7** | Boletales | 22 |
| **17** | Hymenochaetales | 2 |  |  | **8** | Corticiales | 15 |
| **18** | Phacidiales | 2 |  |  | **9** | Chaetosphaeriales | 9 |
| **19** | Phaeomoniellales | 2 |  |  | **10** | Pezizales | 7 |
| **20** | Spizellomycetales | 2 |  |  | **11** | Atheliales | 4 |
| **21** | Coniochaetales | 1 |  |  | **12** | Endogonales | 1 |
| **22** | Ophiostomatales | 1 |  |  | **13** | Eurotiales | 1 |
| **23** | Rhizophydiales | 1 |  |  | **14** | Hysterangiales | 1 |
| **24** | Rhytismatales | 1 |  |  |  |  |  |
| **25** | Ustilaginales | 1 |  |  |  | Ascomycota | 54 |
| **26** | Venturiales | 1 |  |  |  | Basidiomycota | 355 |
|  |  |  |  |  |  | Mucoromycota | 1 |
|  | Ascomycota | 270 |  |  |  |  |  |
|  | Basidiomycota | 31 |  |  |  |  |  |
|  | Chytridiomycota | 5 |  |  |  |  |  |
|  | Entorrhizomycota | 3 |  |  |  |  |  |

## Supplementary Table 4. Best subsets model selection for saprotrophic fungal community composition. The lower gray marked model constitutes the one presented in the manuscript. It was chosen as all model variables were significant and for the number of included variables it showed the highest F value. The upper model was marked as there is additionally an increase in explained variance (R^2) of at least 5% for the addition of one variable compared to the best model subset with one variable less. No.: Running number of the model, V1: number of variables included in the model, Int: Intercept, Ctot: total carbon content, CN: carbon to nitrogen ratio, CEC: effective cation exchange capacity, BS: base saturation, SWC: soil water content.

| **No** | **V1** | **F** | **F.pval** | **R2** | **pval** | **Int** | **Plot** | **location** | **pH** | **Ctot** | **CN** | **CEC** | **BS** | **SWC** |
| --- | --- | --- | --- | --- | --- | --- | --- | --- | --- | --- | --- | --- | --- | --- |
| 1 | 1 | 1556 | 1.00E-04 | 0.33 | 1.00E-04 | 1 | 1.00E-04 | NA | NA | NA | NA | NA | NA | NA |
| 2 | 1 | 1478 | 1.00E-04 | 0.32 | 1.00E-04 | 1 | NA | 1.00E-04 | NA | NA | NA | NA | NA | NA |
| 3 | 1 | 883 | 1.00E-04 | 0.22 | 1.00E-04 | 0.882 | NA | NA | NA | NA | 1.00E-04 | NA | NA | NA |
| 4 | 2 | 1258 | 1.00E-04 | 0.44 | 1.00E-04 | 1 | 1.00E-04 | NA | NA | NA | 1.00E-04 | NA | NA | NA |
| 5 | 2 | 1219 | 1.00E-04 | 0.44 | 1.00E-04 | 1 | NA | 1.00E-04 | NA | NA | 1.00E-04 | NA | NA | NA |
| 6 | 2 | 1212 | 1.00E-04 | 0.43 | 1.00E-04 | 1 | 1.00E-04 | NA | 1.00E-04 | NA | NA | NA | NA | NA |
| 7 | 3 | 1043 | 1.00E-04 | **0.50** | 1.00E-04 | 1 | 1.00E-04 | NA | NA | NA | 1.00E-04 | NA | 1.00E-04 | NA |
| 8 | 3 | 1003 | 1.00E-04 | 0.49 | 1.00E-04 | 1 | 1.00E-04 | NA | 1.00E-04 | NA | 1.00E-04 | NA | NA | NA |
| 9 | 3 | 984 | 1.00E-04 | 0.48 | 1.00E-04 | 1 | NA | 1.00E-04 | NA | NA | 1.00E-04 | NA | 1.00E-04 | NA |
| 10 | 4 | 838 | 1.00E-04 | 0.52 | 1.00E-04 | 1 | 1.00E-04 | NA | NA | NA | 1.00E-04 | 0.0011 | 1.00E-04 | NA |
| 11 | 4 | 832 | 1.00E-04 | 0.51 | 1.00E-04 | 1 | 1.00E-04 | NA | NA | 6.00E-04 | 1.00E-04 | NA | 1.00E-04 | NA |
| 12 | 4 | 816 | 1.00E-04 | 0.51 | 1.00E-04 | 1 | 1.00E-04 | NA | 1.00E-04 | NA | 1.00E-04 | 3.00E-04 | NA | NA |
| 13 | 5 | 690 | 1.00E-04 | 0.52 | 1.00E-04 | 1 | 1.00E-04 | 0.0011 | NA | NA | 1.00E-04 | 7.00E-04 | 1.00E-04 | NA |
| 14 | 5 | 690 | 1.00E-04 | 0.52 | 1.00E-04 | 1 | 1.00E-04 | NA | NA | 3.00E-04 | 1.00E-04 | NA | 1.00E-04 | 0.0054 |
| 15 | 5 | 687 | 1.00E-04 | 0.52 | 1.00E-04 | 1 | 1.00E-04 | NA | NA | NA | 1.00E-04 | 0.0014 | 1.00E-04 | 0.0242 |
| 16 | 6 | 598 | 1.00E-04 | 0.53 | 1.00E-04 | 1 | 1.00E-04 | 2.00E-04 | NA | 7.00E-04 | 1.00E-04 | NA | 1.00E-04 | 0.0019 |
| 17 | 6 | 594 | 1.00E-04 | 0.53 | 1.00E-04 | 1 | 1.00E-04 | 3.00E-04 | NA | NA | 1.00E-04 | 0.002 | 1.00E-04 | 0.0063 |
| 18 | 6 | 587 | 1.00E-04 | 0.53 | 1.00E-04 | 1 | 1.00E-04 | NA | NA | 0.0129 | 1.00E-04 | 0.0324 | 1.00E-04 | 0.0123 |
| 19 | 7 | 522 | 1.00E-04 | **0.54** | 1.00E-04 | 1 | 1.00E-04 | 2.00E-04 | NA | 0.0149 | 1.00E-04 | 0.0477 | 1.00E-04 | 0.0027 |
| 20 | 7 | 516 | 1.00E-04 | 0.53 | 1.00E-04 | 1 | 1.00E-04 | 2.00E-04 | 0.203 | 3.00E-04 | 1.00E-04 | NA | 0.0014 | 0.0023 |
| 21 | 7 | 513 | 1.00E-04 | 0.53 | 1.00E-04 | 1 | 1.00E-04 | 3.00E-04 | 0.1494 | NA | 1.00E-04 | 0.0019 | 0.0083 | 0.0087 |
| 22 | 8 | 460 | 1.00E-04 | 0.54 | 1.00E-04 | 1 | 1.00E-04 | 5.00E-04 | 0.1501 | 0.0147 | 1.00E-04 | 0.0397 | 0.0039 | 0.0037 |

| **No** | **V1** | **F** | **F.pval** | **R2** | **pval** | **Int** | **Plot** | **location** | **Tree** | **Ctot** | **CN** |
| --- | --- | --- | --- | --- | --- | --- | --- | --- | --- | --- | --- |
| 1 | 1 | 308.06 | 1.00E-04 | 0.12 | 1.00E-04 | 0.9999 | NA | NA | NA | NA | 1.00E-04 |
| 2 | 1 | 265.93 | 1.00E-04 | 0.11 | 1.00E-04 | 1 | 1.00E-04 | NA | NA | NA | NA |
| 3 | 1 | 232.92 | 1.00E-04 | 0.10 | 1.00E-04 | 1 | NA | NA | 1.00E-04 | NA | NA |
| 4 | 2 | 291.93 | 1.00E-04 | **0.21** | 1.00E-04 | 1 | NA | NA | 1.00E-04 | NA | 1.00E-04 |
| 5 | 2 | 255.09 | 1.00E-04 | 0.19 | 1.00E-04 | 1 | 1.00E-04 | NA | NA | NA | 1.00E-04 |
| 6 | 2 | 236.75 | 1.00E-04 | 0.18 | 1.00E-04 | 1 | NA | 1.00E-04 | NA | NA | 1.00E-04 |
| 7 | 3 | 215.38 | 1.00E-04 | **0.23** | 1.00E-04 | 1 | NA | 3.00E-04 | 1.00E-04 | NA | 1.00E-04 |
| 8 | 3 | 213.20 | 1.00E-04 | 0.22 | 1.00E-04 | 1 | 1.00E-04 | NA | 1.00E-04 | NA | 1.00E-04 |
| 9 | 3 | 201.29 | 1.00E-04 | 0.21 | 1.00E-04 | 1 | NA | NA | 1.00E-04 | 0.1423 | 1.00E-04 |
| 10 | 4 | 165.61 | 1.00E-04 | 0.23 | 1.00E-04 | 1 | NA | 4.00E-04 | 1.00E-04 | 0.1844 | 1.00E-04 |
| 11 | 4 | 164.74 | 1.00E-04 | 0.23 | 1.00E-04 | 1 | 1.00E-04 | NA | 1.00E-04 | 0.1515 | 1.00E-04 |
| 12 | 4 | 162.57 | 1.00E-04 | 0.23 | 1.00E-04 | 1 | 0.3434 | 0.1789 | 1.00E-04 | NA | 1.00E-04 |
| 13 | 5 | 133.51 | 1.00E-04 | 0.23 | 1.00E-04 | 1 | 0.2849 | 0.2235 | 1.00E-04 | 0.1834 | 1.00E-04 |

**Supplementary Table 5.** Best subsets model selection for fungal plant pathogen community composition. The lower gray marked model constitutes the one presented in the manuscript. It was chosen as all model variables were significant and for the number of variables included it showed the highest F value. The upper model is marked as there was also an increase in explained variance (R^2) of at least 5% for the addition of one variable compared to the best model subset with one variable less. No.: Running number of the model, V1: number of variables included in the model, Int: Intercept, Ctot: total carbon content, CN: carbon to nitrogen ratio, CEC: effective cation exchange capacity, BS: base saturation, SWC: soil water content.

**Supplementary Table 6.** Best subsets model selection for ectomycorrhizal fungi community composition. The lower gray marked model constitutes the one presented in the manuscript. It was chosen as all model variables were significant and for the number of variables included it showed the highest F value. The upper model is marked as there was also an increase in explained variance (R^2) of at least 5% for the addition of one variable compared to the best model subset with one variable less. No.: Running number of the model, V1: number of variables included in the model, Int: Intercept, Ctot: total carbon content, CN: carbon to nitrogen ratio, CEC: effective cation exchange capacity, BS: base saturation, SWC: soil water content.

| **No** | **V1** | **F** | **F.pval** | **R2** | **pval** | **Int** | **Plot** | **location** | **Sample_Tree** | **Tree Myco** | **EcM_ab** | **EcM_richness** |
| --- | --- | --- | --- | --- | --- | --- | --- | --- | --- | --- | --- | --- |
| 1 | 1 | 290.94 | 1.00E-04 | 0.14 | 1.00E-04 | 1 | 1.00E-04 | NA | NA | NA | NA | NA |
| 2 | 1 | 245.56 | 1.00E-04 | 0.12 | 1.00E-04 | 1 | NA | 1.00E-04 | NA | NA | NA | NA |
| 3 | 1 | 171.37 | 1.00E-04 | 0.09 | 1.00E-04 | 0.1066 | NA | NA | NA | 1.00E-04 | NA | NA |
| 4 | 2 | 275.54 | 1.00E-04 | **0.24** | 1.00E-04 | 1 | 1.00E-04 | NA | NA | 1.00E-04 | NA | NA |
| 5 | 2 | 247.28 | 1.00E-04 | 0.22 | 1.00E-04 | 1 | NA | 1.00E-04 | NA | 1.00E-04 | NA | NA |
| 6 | 2 | 181.87 | 1.00E-04 | 0.17 | 1.00E-04 | 1 | 1.00E-04 | NA | NA | NA | 0.0013 | NA |
| 7 | 3 | 194.15 | 1.00E-04 | 0.25 | 1.00E-04 | 1 | 1.00E-04 | NA | NA | 1.00E-04 | 0.0452 | NA |
| 8 | 3 | 193.50 | 1.00E-04 | 0.25 | 1.00E-04 | 1 | 1.00E-04 | NA | NA | 1.00E-04 | NA | 0.016 |
| 9 | 3 | 187.17 | 1.00E-04 | 0.24 | 1.00E-04 | 1 | 1.00E-04 | NA | 0.003 | 1.00E-04 | NA | NA |
| 10 | 4 | 148.76 | 1.00E-04 | 0.25 | 1.00E-04 | 1 | 1.00E-04 | 0.0452 | NA | 1.00E-04 | 0.03 | NA |
| 11 | 4 | 148.39 | 1.00E-04 | 0.25 | 1.00E-04 | 1 | 1.00E-04 | NA | 0.0022 | 1.00E-04 | 0.0392 | NA |
| 12 | 4 | 148.32 | 1.00E-04 | 0.25 | 1.00E-04 | 1 | 3.00E-04 | 0.0454 | NA | 1.00E-04 | NA | 0.0112 |
| 13 | 5 | **121.32** | 1.00E-04 | **0.26** | 1.00E-04 | 1 | 1.00E-04 | 0.0405 | 0.0024 | 1.00E-04 | 0.0285 | NA |
| 14 | 5 | **120.98** | 1.00E-04 | **0.26** | 1.00E-04 | 1 | 3.00E-04 | 0.0406 | 0.0024 | 1.00E-04 | NA | 0.0092 |
| 15 | 5 | 120.75 | 1.00E-04 | 0.25 | 1.00E-04 | 1 | 2.00E-04 | 0.0313 | NA | 1.00E-04 | 0.1838 | 0.1034 |
| 16 | 6 | 102.60 | 1.00E-04 | 0.26 | 1.00E-04 | 1 | 1.00E-04 | 0.039 | 0.0019 | 1.00E-04 | 0.1752 | 0.0985 |

**Supplementary Table 7.** Best subsets model selection for arbuscular mycorrhizal fungal community composition. The lower gray marked model constitutes the one presented in the manuscript. It was chosen as all model variables were significant and for the number of variables included it showed the highest F value. The upper model is marked as there was also an increase in explained variance (R^2) of at least 5% for the addition of one variable compared to the best model subset with one variable less. No.: Running number of the model, V1: number of variables included in the model, Int: Intercept, Ctot: total carbon content, CN: carbon to nitrogen ratio, CEC: effective cation exchange capacity, BS: base saturation, SWC: soil water content.

| **No** | **V1** | **F** | **F.pval** | **R2** | **pval** | **Int** | **Plot** | **location** | **Tree** | **pH** | **Ctot** | **CN** | **CEC** | **BS** |
| --- | --- | --- | --- | --- | --- | --- | --- | --- | --- | --- | --- | --- | --- | --- |
| 1 | 1 | 705.65 | 1.00E-04 | 0.22 | 1.00E-04 | 0.3903 | NA | NA | NA | 1.00E-04 | NA | NA | NA | NA |
| 2 | 1 | 647.55 | 1.00E-04 | 0.21 | 1.00E-04 | 0.8591 | NA | NA | NA | NA | NA | 1.00E-04 | NA | NA |
| 3 | 1 | 588.33 | 1.00E-04 | 0.19 | 1.00E-04 | 1 | NA | 1.00E-04 | NA | NA | NA | NA | NA | NA |
| 4 | 2 | 607.70 | 1.00E-04 | 0.33 | 1.00E-04 | 1 | NA | 1.00E-04 | NA | 1.00E-04 | NA | NA | NA | NA |
| 5 | 2 | 605.46 | 1.00E-04 | 0.33 | 1.00E-04 | 1 | 1.00E-04 | NA | NA | 1.00E-04 | NA | NA | NA | NA |
| 6 | 2 | 585.57 | 1.00E-04 | 0.32 | 1.00E-04 | 1 | NA | 1.00E-04 | NA | NA | NA | 1.00E-04 | NA | NA |
| 7 | 3 | 510.16 | 1.00E-04 | 0.38 | 1.00E-04 | 1 | NA | 1.00E-04 | NA | 2.00E-04 | NA | 1.00E-04 | NA | NA |
| 8 | 3 | 509.51 | 1.00E-04 | 0.38 | 1.00E-04 | 1 | 1.00E-04 | NA | NA | NA | NA | 1.00E-04 | NA | 1.00E-04 |
| 9 | 3 | 508.00 | 1.00E-04 | 0.38 | 1.00E-04 | 1 | NA | 1.00E-04 | NA | NA | NA | 1.00E-04 | NA | 1.00E-04 |
| 10 | 4 | 436.50 | 1.00E-04 | 0.41 | 1.00E-04 | 1 | NA | 1.00E-04 | NA | 2.00E-04 | NA | 2.00E-04 | 0.0011 | NA |
| 11 | 4 | 433.89 | 1.00E-04 | 0.41 | 1.00E-04 | 1 | 1.00E-04 | NA | NA | NA | NA | 1.00E-04 | 0.0015 | 1.00E-04 |
| 12 | 4 | 433.53 | 1.00E-04 | 0.41 | 1.00E-04 | 1 | 1.00E-04 | NA | NA | 1.00E-04 | NA | 2.00E-04 | 7.00E-04 | NA |
| 13 | 5 | 379.32 | 1.00E-04 | **0.43** | 1.00E-04 | 1 | NA | 1.00E-04 | 1.00E-04 | 1.00E-04 | NA | 2.00E-04 | 0.0015 | NA |
| 14 | 5 | 371.93 | 1.00E-04 | 0.43 | 1.00E-04 | 1 | NA | 1.00E-04 | 2.00E-04 | NA | NA | 1.00E-04 | 0.0014 | 3.00E-04 |
| 15 | 5 | 368.84 | 1.00E-04 | 0.43 | 1.00E-04 | 1 | 1.00E-04 | NA | 1.00E-04 | 2.00E-04 | NA | 1.00E-04 | 8.00E-04 | NA |
| 16 | 6 | 320.02 | 1.00E-04 | 0.44 | 1.00E-04 | 1 | NA | 1.00E-04 | 1.00E-04 | 0.0421 | NA | 1.00E-04 | 0.0012 | 0.172 |
| 17 | 6 | 319.34 | 1.00E-04 | 0.44 | 1.00E-04 | 1 | NA | 1.00E-04 | 1.00E-04 | 1.00E-04 | 0.2156 | 1.00E-04 | 0.0054 | NA |
| 18 | 6 | 316.94 | 1.00E-04 | 0.43 | 1.00E-04 | 1 | 0.3477 | 0.0054 | 2.00E-04 | 1.00E-04 | NA | 2.00E-04 | 0.0016 | NA |
| 19 | 7 | 277.21 | 1.00E-04 | 0.44 | 1.00E-04 | 1 | NA | 1.00E-04 | 1.00E-04 | 0.0325 | 0.2133 | 1.00E-04 | 0.0061 | 0.1659 |
| 20 | 7 | 275.34 | 1.00E-04 | 0.44 | 1.00E-04 | 1 | 0.2736 | 0.0066 | 1.00E-04 | 0.048 | NA | 1.00E-04 | 0.0018 | 0.1558 |
| 21 | 7 | 274.40 | 1.00E-04 | 0.44 | 1.00E-04 | 1 | 0.3712 | 0.0029 | 1.00E-04 | 1.00E-04 | 0.2293 | 1.00E-04 | 0.0044 | NA |
| 22 | 8 | 243.43 | 1.00E-04 | 0.44 | 1.00E-04 | 1 | 0.282 | 0.0063 | 1.00E-04 | 0.0392 | 0.2152 | 1.00E-04 | 0.0047 | 0.146 |
